# Analysis of gene expression during neurite outgrowth and regeneration

**DOI:** 10.1186/1471-2202-8-100

**Published:** 2007-11-23

**Authors:** Moriah L Szpara, Karen Vranizan, Yu Chuan Tai, Corey S Goodman, Terence P Speed, John Ngai

**Affiliations:** 1 Department of Molecular and Cell Biology, University of California, Berkeley, California, USA; 2 Helen Wills Neuroscience Institute, University of California, Berkeley, California, USA; 3 Functional Genomics Laboratory, University of California, Berkeley, California, USA; 4 Division of Biostatistics, University of California, Berkeley, California, USA; 5 Department of Statistics, University of California, Berkeley, California, USA

## Abstract

**Background:**

The ability of a neuron to regenerate functional connections after injury is influenced by both its intrinsic state and also by extrinsic cues in its surroundings. Investigations of the transcriptional changes undergone by neurons during *in vivo *models of injury and regeneration have revealed many transcripts associated with these processes. Because of the complex milieu of interactions *in vivo*, these results include not only expression changes directly related to regenerative outgrowth and but also unrelated responses to surrounding cells and signals. *In vitro *models of neurite outgrowth provide a means to study the intrinsic transcriptional patterns of neurite outgrowth in the absence of extensive extrinsic cues from nearby cells and tissues.

**Results:**

We have undertaken a genome-wide study of transcriptional activity in embryonic superior cervical ganglia (SCG) and dorsal root ganglia (DRG) during a time course of neurite outgrowth *in vitro*. Gene expression observed in these models likely includes both developmental gene expression patterns and regenerative responses to axotomy, which occurs as the result of tissue dissection. Comparison across both models revealed many genes with similar gene expression patterns during neurite outgrowth. These patterns were minimally affected by exposure to the potent inhibitory cue Semaphorin3A, indicating that this extrinsic cue does not exert major effects at the level of nuclear transcription. We also compared our data to several published studies of DRG and SCG gene expression in animal models of regeneration, and found the expression of a large number of genes in common between neurite outgrowth *in vitro *and regeneration *in vivo*.

**Conclusion:**

Many gene expression changes undergone by SCG and DRG during *in vitro *outgrowth are shared between these two tissue types and in common with *in vivo *regeneration models. This suggests that the genes identified in this *in vitro *study may represent new candidates worthy of further study for potential roles in the therapeutic regrowth of neuronal connections.

## Background

Neuronal development, as well as neuronal response to injury, depend on both intrinsic programs of gene expression and on extrinsic cues from the surrounding environment. Understanding the balance between these two, and potentially influencing it, are the focus of current strategies to improve neuronal regeneration after injury [1]. That the intrinsic state of a neuron can be manipulated to improve regeneration has been well-demonstrated in dorsal root ganglia (DRG) neurons, where neurite outgrowth in response to spinal nerve injury is significantly improved by a preceding peripheral nerve lesion [2-5]. This ability of intrinsic changes to influence neurite outgrowth has been further demonstrated by transgenic expression of genes such as cytoskeleton-associated protein 23 (CAP23), growth associated protein 43 (GAP43), small proline-rich repeat protein 1A (Sprr1A), and activating transcription factor 3 (Atf3) [6-10].

In addition to intrinsic effects, the extrinsic environment also vastly influences outgrowth ability [11,12]. For example, neurite regrowth is affected by injury-induced inhibitory factors such as Semaphorins, Nogo, and myelin-associated glycoprotein, as well as reactive microglia and fibrous scar tissue [12-15]. Grafts of peripheral nervous system (PNS) tissue at sites of central nervous system (CNS) lesion can improve neurite growth across an otherwise inhibitory scar, and much research has been focused on distinguishing the growth-permissive and growth-inhibitory aspects of these two [16-18].

Measuring changes in neuronal gene expression in models of regeneration has allowed for a combinatorial readout of both intrinsic and extrinsic factors, since many extrinsic cues elicit intracellular signals that affect transcription [19-26]. However the complexity of *in vivo *models means that responses that causally affect a neuron's regeneration are observed simultaneously with dispensable secondary or tertiary effects of the environment and signaling from other affected cells. *In vitro *models provide an opportunity to address this, by allowing for tighter experimental control of the extrinsic environment while still allowing measurement of intrinsic transcriptional activity.

In the present study, we have used two *in vitro *model systems, those of superior cervical ganglia (SCG) and DRG, to study intrinsic transcriptional activity during neurite outgrowth. Explanting these tissues provides the dual conditions of axotomy, which mirrors *in vivo *regeneration models, and a controlled extracellular environment, where we can observe gene expression changes during neurite outgrowth in the absence of signaling from multiple guidance factors and injury-induced signals *in vivo*. We compared these effects to published data on genes associated with regeneration *in vivo*, and found many areas of commonality. We also attempted to perturb these gene expression patterns with exposure to Semaphorin 3A (Sema3A), a potent extrinsic inhibitory cue that affects both local growth cone morphology and also axoplasmic transport – a means of potentially conveying signals back to the nucleus [27,28]. These data demonstrate the applicability of comparing across *in vitro *and *in vivo *models of neurite outgrowth, and their potential for revealing genes involved in intrinsic patterns of regeneration.

## Results

### Intrinsic patterns of gene expression during neurite outgrowth *in vitro*

We wished to determine the transcriptional profiles of neurons undergoing neurite outgrowth *in vitro*. We were particularly interested in finding genes whose expression is generally associated with the process of neurite outgrowth, rather than with cell type-specific effects. Thus, in order to avoid focusing on transcripts unique to one tissue type versus another, we used a comparative strategy to look for effects that were common to two tissue types and therefore more likely to be involved in the general process of neurite outgrowth. While these explants contain multiple cell types, we felt this was preferable to the more disruptive conditions required to dissociate neurons or obtain a pure neuron population. To this end, we monitored gene expression in cultured explants from SCG and DRG using DNA microarrays.

We initiated our studies by culturing embryonic day 13 (E13) mouse SCG *in vitro *and harvesting tissue for RNA isolation at time points from 2 to 65 hours. Time points were selected to detect both fast, short-term responses (2, 5 and 12 hours), as well as sustained, long-term changes (24, 40, and 65 hours). Samples were hybridized to Affymetrix MG-U74v2 A and B microarrays, with RNA from acutely dissected explants serving as a baseline reference. In SCG, 5,097 probesets were observed to change 1.5 fold or greater over time. To test if these changes were significantly different from the null hypothesis (no change over time), we used a polynomial regression of the maximum of quadratic order. This analysis revealed that 1,728 probesets were significantly changed (p ≤ 0.05) during neurite outgrowth by SCG.

We followed these experiments with a parallel analysis of a more heterogeneous tissue type, the DRG, which is more frequently used than SCG for *in vivo *studies of neurite regeneration. Cervical and upper thoracic DRG from E12 embryos were cultured with NGF (the same trophic support as in SCG cultures) and harvested at time points from 2 to 40 hours. Using Affymetrix MOE 430A microarrays, 4,860 probesets were observed to change 1.5 fold or more during neurite outgrowth in cultured DRG, with 2,632 changes of p ≤ 0.05 by quadratic statistical analysis.

**Figure 1:**
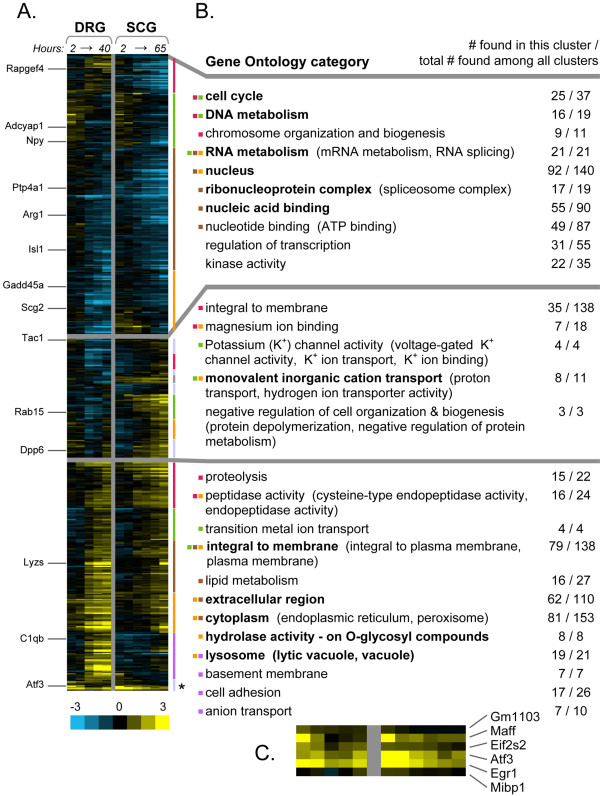
**HOPACH clustering and GenMAPP annotation of genes changed in both DRG and SCG during neurite outgrowth *in vitro***. **(A) **The cluster displays the averaged Affymetrix microarray data for 5 time points of DRG outgrowth (2, 5, 12, 24, and 40 hours) and 6 time points of SCG outgrowth (same as DRG plus 65 hour endpoint), for the 712 probe sets found to change more than 1.5 fold (p ≤ 0.05) in both datasets. Genes annotated along the left column are those also found in two or more of the *in vivo *regeneration studies (see Figure 2). Yellow indicates increased expression or probe intensity relative to the acutely dissected reference pool, while blue indicates decreased expression (log_2 _scale). Horizontal gray bars separate the first level clusters, and colored vertical bars along the right indicate the next level of sub-clusters. **(B) **MAPPFinder annotations of Gene Ontology categories represented in the first-level clusters, along with number of genes found in this cluster relative to the total number found in all three clusters for each category (all Z-scores > 2 and permuted-p ≤ 0.05). Terms in parentheses show related categories that were also significant. Terms in bold have a p-value ≤ 0.05 after adjustment for multiple hypothesis testing. In cases where annotations for first-level clusters also appeared in MAPPFinder annotation of the next level sub-clusters (with Z-scores > 2, permuted-p ≤ 0.05), those localizations to sub-clusters are indicated by an appropriately colored square. **(C) **Six nucleic-acid binding genes were detected by GenMAPP in a sub-cluster of (A) (marked by asterisk). These genes peak temporally earlier than most others in cluster 3. Ratio of expression change over time for these genes is color-coded as in (A).

The transcripts commonly affected during neurite outgrowth by both these model neuron populations may represent those most relevant to neurite outgrowth or regeneration in general. Thus, we combined the SCG and DRG microarray data by identifying 11,268 matching probesets from the two types of Affymetrix microarrays used for these experiments (MG-U74v2 A and B for SCG and MOE430A for DRG). 712 matched probesets were changed significantly at least 1.5 fold (p ≤ 0.05). To visualize these gene expression profiles, a clustering algorithm called Hierarchical Ordered Partitioning and Collapsing Hybrid (HOPACH) was used (Figure 1) [29]. HOPACH is similar to traditional hierarchical clustering, but also provides statistically set cluster divisions at each sub-level [30]. The data are visualized as a dual time-course, with the DRG samples over time followed by the SCG samples over time (Figure 1A). The first-level HOPACH clusters (horizontal gray lines, Figure 1A) group genes with common regulation patterns in both DRG and SCG (e.g., up-regulated in both or down-regulated in both), as well as those with more divergent patterns.

We wished to determine the biological functions of these gene clusters. The MAPPFinder function of GenMAPP was used to test for significant localization of particular biological functions (listed as Gene Ontology (GO) terms) to each of the main HOPACH clusters [31-34]. GO terms over-represented in each cluster, as compared to their presence in the whole dataset, are shown in Figure 1B (p ≤ 0.05). Significant down-regulation, by both SCG and DRG, was found for genes involved in cell division and nuclear metabolism (1^st ^cluster, Figure 1). Common upregulation (3^rd ^cluster, Figure 1) was observed for genes associated with extracellular matrix, basement membrane, and cell adhesion, suggesting remodeling of the local environment to allow axon outgrowth. Increases in both SCG and DRG of genes involved in proteolysis and lysosome function echo previous observations of increases in these gene families after neuronal injury [25,35]. Finally, neurite outgrowth relies on the increased production of both membrane and cytoplasmic components [36], as can be observed in the large numbers of genes in the commonly upregulated group (3^rd ^cluster, Figure 1), where genes involved in lipid metabolism and cytoplasmic components are localized.

In addition to looking at DRG and SCG changes together, the data for each time course of neurite outgrowth were clustered and annotated separately (see Additional file 1 – Additional Figures 2 and 3). One of the unique categories identified in DRG but not SCG is the late induction of apoptosis-related genes. This is not surprising, since apoptosis is normally underway in DRG neurons of this age, while SCG neurons undergo apoptosis *in vivo *at a later age [37,38]. In addition, while most SCG neurons are NGF-responsive at this time, DRG are more heterogeneous and include both NGF- and neurotrophin 3-responsive neurons, of which the latter may undergo cell death in these cultures [39-42]. MAPPFinder annotation of these clusters thus confirms previously observed differences in their developmental programs, and demonstrates that functions known to be involved in neurite outgrowth are represented in these data.

We also wanted to uncover novel biological associations in these data. As one approach to this, we examined the next smaller level of HOPACH clusters. Most significant biological functions localized in these smaller clusters were also represented in the larger main clusters, as shown by the vertical colored bars and matching colored boxes in the GO term list in Figure 1. However one example of novel biological associations found at the sub-cluster level is demonstrated by a group of genes at the bottom of Figure 1A. This sub-cluster contains twelve genes, of which six are associated by GenMAPP with nucleic acid binding (permuted P = 0.002): Atf3, early growth response 1 (Egr1/Krox-24/Zif-268/NGFIA), eukaryotic translation initiation factor 2 (Eif2s2), Gm1103 (or Zbtb2), musculoaponeurotic fibrosarcoma oncogene family protein F (Maff), and c-myc intron 1 binding protein (Mibp1) (Figure 1C). The expression of these genes peaks earlier than most other genes in cluster 3, suggesting a potential for temporal regulation. Atf3 upregulation has been previously noted after injury, and its regeneration-promoting ability has been recently demonstrated *in vivo *by Seijffers et al (2007) [7,19,21,43]. Egr1 is a multi-functional transcription regulator in both neuronal and non-neuronal cells. In the nervous system, it is associated with plasticity and synaptic activity, although a transient activation after neuronal injury has been noted [44,45]. Maff has recently been described as part of a negative downstream regulatory loop after activation of Egr1 [46]. Although Mibp1 has been previously found to be neuronally expressed [47], neither Mibp1, Eif2s2, Gm1103, nor Maff have been previously linked to regeneration. However their close association with Atf3 and similar temporal expression suggests them as good candidates for a role in neurite outgrowth and regeneration.

### Relating gene expression patterns of growth *in vitro *to regeneration *in vivo*

**Figure 2:**
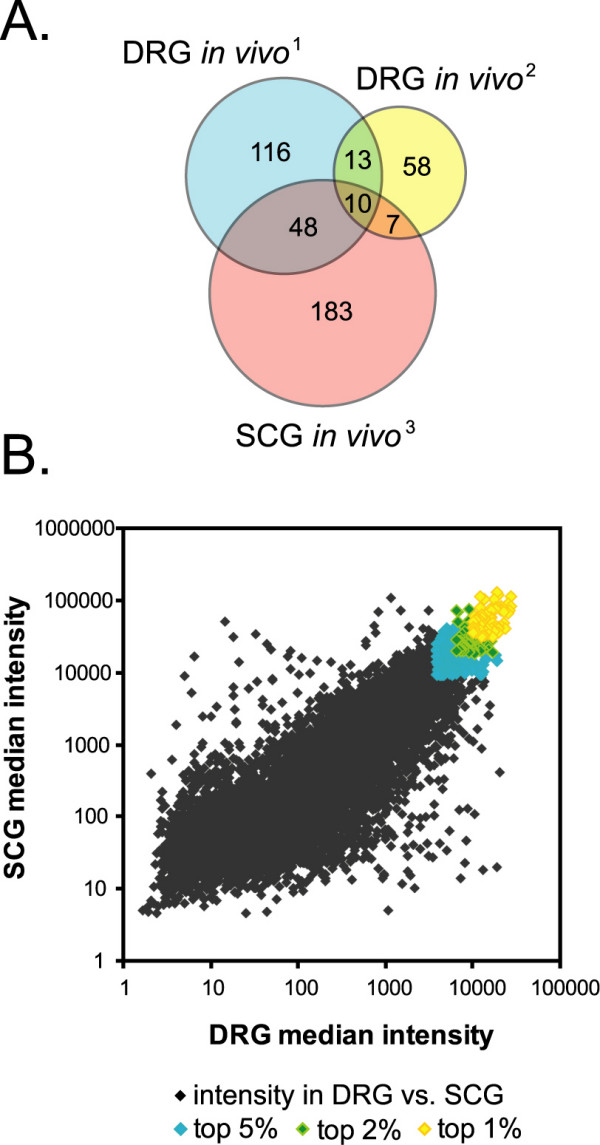
**Meta-analysis of genes changed during *in vivo *models of regeneration**. **(A) **Venn diagram of genes affected in common across *in vivo *regeneration models. Data from three published studies of *in vivo *regeneration after peripheral axotomy in adult rats were compared to each other. Those genes found by two or more of these studies (shaded regions of overlap, n = 78) are also noted on the clusters found in Figure 1 and in Additional file 1 (Additional Figures 2 and 3). All genes were mapped to a common Entrez Gene ID, which is listed in Additional file 2 along with associated data. **(B) **Scatter plot of median intensity in both DRG and SCG for each unique Entrez gene found in this study. Probesets were collapsed to select only one unique Entrez ID per gene, so that genes with multiple probesets on the array would not skew the data. Blue, green, and yellow points highlight genes whose intensity of expression are respectively in the top 5%, 2%, and 1% intersection of all DRG- and SCG-expressed genes. Further annotation of these highly-expressed transcripts is found in Figure 3. DRG *in vivo*^1^, rat gene regulated 3 days after sciatic nerve axotomy (n = 187) [21]. DRG *in vivo*^2^, rat gene regulated 2 days after sciatic nerve axotomy (n = 88) [23]. SCG *in vivo*^3^, rat gene regulated 2 days after post-ganglionic nerve axotomy (n = 230) [19].

To understand how the transcriptional patterns of neurite outgrowth in cultured DRG and SCG relate to regeneration of adult ganglia, these data were compared to several published studies of adult DRG and SCG regeneration *in vivo*. For this meta-analysis, three recently published studies with large datasets were selected, and the time point from each study that most closely matched the other available data was used. From Costigan et al., the data included 187 unique genes significantly regulated at least 1.5 fold in lumbar DRG at three days after sciatic nerve axotomy [21]. In Xiao et al., 88 genes were significantly regulated at least 2 fold in lumbar DRG at two days after sciatic nerve axotomy [23]. Finally, the data from Boeshore et al. provided 248 genes significantly regulated at least 2 fold in SCG at two days after axotomy of the internal and external carotid nerves [19]. A total of 78 genes are found in common among at least two of these three studies of neurite regeneration *in vivo *(Figure 2A, see also Additional file 2 – Meta-analysis table), of which 66 are found among the probesets in our data. Of the 66 genes found in the DRG-SCG *in vitro *dataset, 34 genes were also significantly changed during *in vitro *outgrowth of one or both explant types.

Our data provide an opportunity to look at the behavior of this group of previously identified regeneration-associated genes in two common *in vitro *models of neurite outgrowth. At the time of dissection both DRG and SCG have extended significant nerve projections (E12 for DRG and E13 for SCG) [48-52]. Therefore these embryonic *in vitro *culture models encompass both ongoing developmental gene expression as well as effects of axotomy. We localized the 34 genes commonly affected during *in vivo *regeneration models and *in vitro *outgrowth models on the cluster diagrams described above (annotated in Figure 1A and Additional file 1 – Additional Figures 2 and 3; see also Additional file 2 – Meta-analysis and Abbreviations tables). The localization of these changed genes on the cluster diagrams provides insights into which of these *in vivo *regeneration-associated genes may be part of an intrinsic outgrowth program. For instance, down-regulation of the transcription factor Isl1 and up-regulation of the catabolic enzyme lysozyme have both been previously noted during DRG and SCG *in vivo *regeneration, and these genes behave similarly during embryonic neurite outgrowth [19,21,23,53]. This suggests that their functions may be truly intrinsic to this process. However genes such as neuropeptide Y (Npy), growth arrest and DNA damage inducible protein 45 (Gadd45A), arginase 1 (Arg1), and protein tyrosine phosphatase 4a1, all of which are upregulated during *in vivo *regeneration, are down-regulated over time during outgrowth by embryonic SCG and DRG [19,21-23,53-56]. This suggests that upregulation of these genes may not be an absolute requirement for neurite outgrowth or regeneration. Thus the present data not only suggests additional outgrowth-associated genes, but may also suggest which *in vivo *changes are intrinsic to neurite extension, and which may be the result of the extrinsic environment or synaptic interactions.

In addition to these 34 genes changed both *in vivo *and *in vitro*, there were also 32 regeneration-associated genes present but not changed significantly *in vitro *(see Additional file 2 – Meta-analysis table). These 32 genes are generally of low intensity, with a median intensity value of 5.1 in DRG and 5.65 in SCG, as compared to a median intensity of 8.4 in DRG or 9.1 in SCG for the 34 regeneration- and *in vitro *outgrowth-associated genes. These 32 genes include those functioning mainly in mature neurons (such as galanin and receptors for benzodiazepine, serotonin, or glutamate) or in non-neuronal cells (such as glial fibrillary acidic protein (GFAP) or the glial high affinity glutamate transporter Slc1a3). The lack of significant change for these transcripts *in vitro *is difficult to interpret, since low intensity may reflect alternatively active repression or a low expression level in the growing cells.

The two groups of genes from the meta-analysis encompass two possible patterns of gene expression in explants undergoing outgrowth *in vitro *– first, the up- or down-regulation of transcripts from a starting level, and second, the failure to up or down-regulate a given set of transcripts. A third possibility is that genes highly expressed in developing neurons *in vivo *would be required and/or maintained at a high level of expression during outgrowth *in vitro*. These genes would not appear to be upregulated over time, and would thus not be captured by our previous analyses. Since microarray hybridization intensity usually scales with increasing expression levels, we calculated a median intensity value for each gene in the DRG-SCG dataset as a relative measure of expression. The intensity values were plotted against each other, revealing a linear relationship (r = 0.767) (Figure 2B). Thus many genes are expressed at similar levels in both tissues. For instance, within the top 2% highest intensity genes, more than half (71%) are highly expressed in both DRG and SCG.

**Figure 3:**
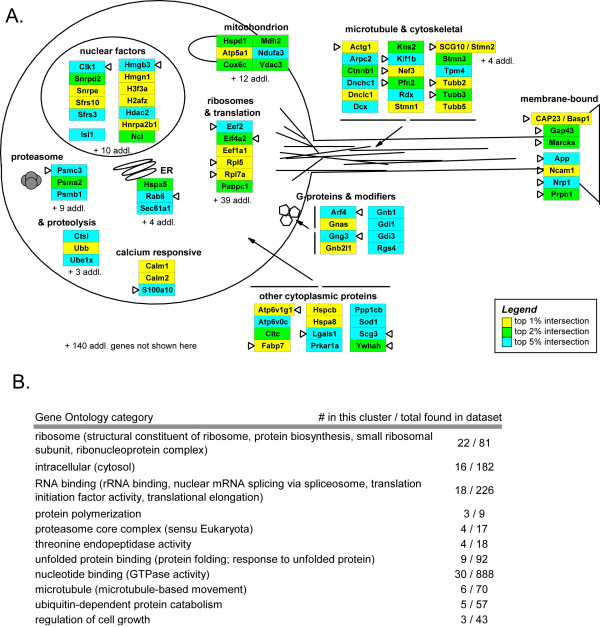
**Gene ontology categories for the genes most highly expressed in both SCG and DRG**. Major gene ontology categories for the genes most highly expressed in both SCG and DRG are colored by probe intensity (same color scheme as Figure 2B), including where available their cellular localization and prior evidence of association with regeneration (triangles). **(A) **Diagram of a neuron with localization of function for 82 of the 325 genes in the top 5% intersection of genes most highly expressed in both DRG and SCG. A triangle to the left of a transcript indicates a gene previously linked to regeneration (see references in text), and triangle to the right of a transcript indicates that another subunit or isoform of this gene was previously linked to regeneration. Gene names are listed in Additional file 2. **(B) **Lists the major Gene Ontology terms found by MAPPFinder for the top 2% intersection of both DRG and SCG expression levels. To the right of each GO term is the number of genes found in this group relative to the rest of the dataset. All observations had a Z-score > 2 and permuted-p ≤ 0.05. Terms in parentheses show related categories that were also significant, and those in bold had a p-value ≤ 0.05 after adjustment for multiple hypothesis testing.

Upon examination of the genes identified in this highest intensity, most highly expressed group of transcripts, we found many genes previously linked to regeneration (see Additional file 2 – Top 5% intersection table). These include several of the best-known regeneration associated genes, including SCG10 (Stathmin 2), Gap43, Cap23 (also known as brain abundant membrane-attached signal protein 1, Basp1), and MARCKS [57-61]. Two other large categories of genes include proteins involved with ribosomal functions or cytoskeletal dynamics, both of which are relevant to the process of neurite outgrowth. To quantify these observations, we used MAPPFinder to assess the over-representation of GO terms among the most highly expressed genes (shown in Figure 3B). Figure 3A illustrates several of these functional groups and notes which transcripts have been previously linked to regeneration [57-61]. Together, the meta-analysis and intensity-analysis confirm that gene expression patterns of neurite outgrowth *in vitro *incorporate many growth-competence genes associated with neurite regeneration *in vivo*, as well as pointing to potentially novel members of these groups for further study.

### Validation of transcriptional patterns associated with neurite outgrowth

The Affymetrix microarray experiments described thus far identify expression changes in SCG and DRG cultures undergoing active neurite outgrowth. To validate these changes, both an alternative microarray platform and quantitative real-time RT-PCR (qPCR) were used. First, we used spotted DNA microarrays of the RIKEN 19 K release set to measure changes in both DRG and SCG samples (for details see Additional file 1 – Additional Methods). These analyses revealed similar patterns of gene expression change as found by Affymetrix arrays (data not shown). We compared the data for transcripts found on both platforms, and of the 2,632 probesets observed to change significantly in DRG during neurite outgrowth, 489 had representative cDNA probes represented in the RIKEN data. This subset of genes displayed an average Spearman correlation of 0.56 (interquartile range (IQR): 0.4, 0.7, 0.9) between the two platforms. For the 1,728 probesets changed significantly in SCG, 395 are represented in the RIKEN data, with an average Spearman correlation of 0.53 (IQR: 0.26, 0.71, 0.89). The positive correlations of these data are visually depicted on a gene-by-gene basis in Additional file 1 – Additional Figures 2A and 3A.

**Figure 4:**
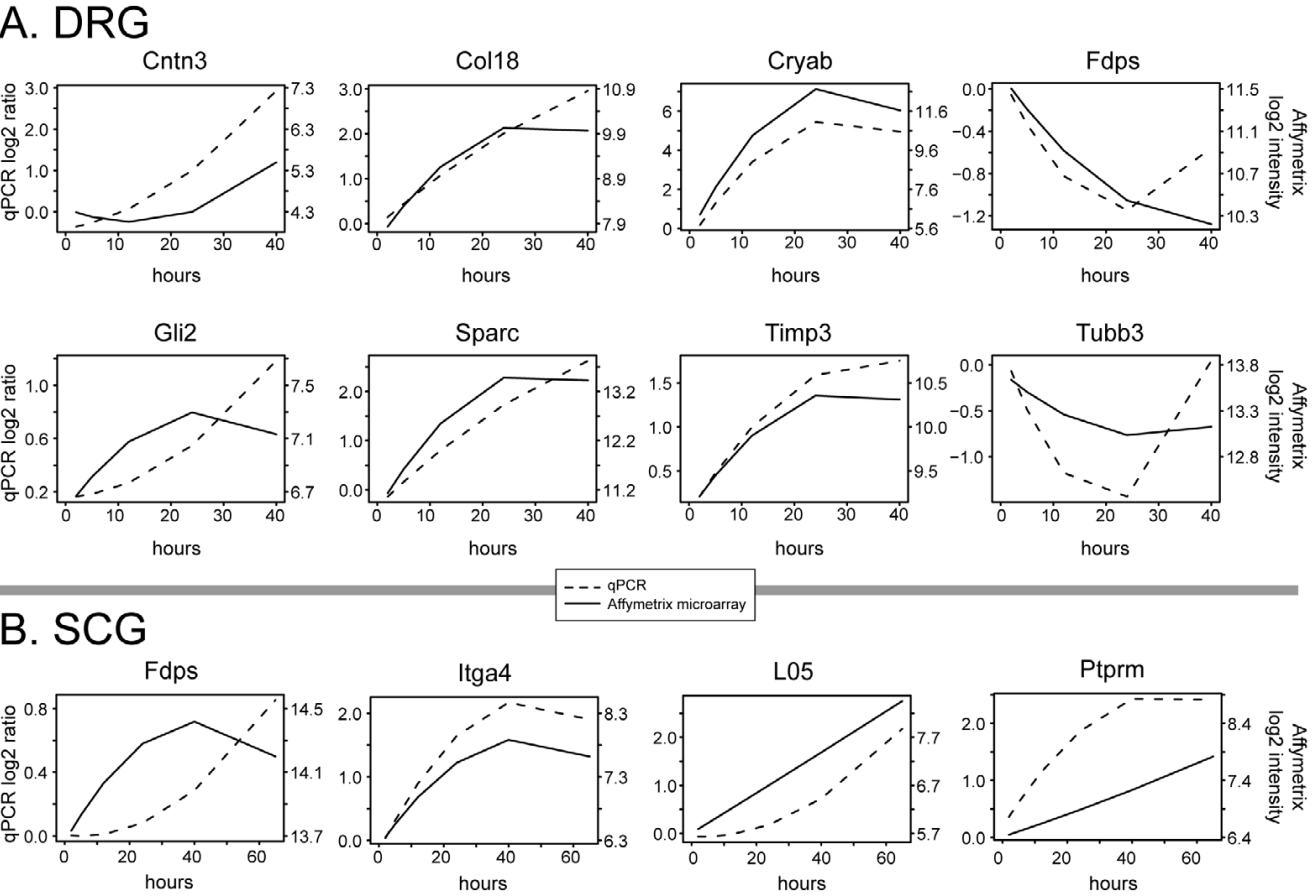
**Comparison of gene expression data by microarray analysis and qPCR**. Time course of gene expression change in DRG **(A) **and in SCG **(B) **for genes with statistically significant change by both microarray and qPCR (p ≤ 0.05; see Tables 1–2 for gene names and details). Solid lines plot the quadratic fit of Affymetrix intensity values over time, while the dashed line represents the quadratic fit of the qPCR log ratios. All data is expressed in a log_2 _scale. qPCR data are normalized to a common reference gene (Wdr4; see Methods for details) and plotted relative to the 2 hour starting point.

We also selected several genes for individual validation using qPCR with dual-labeled fluorescent hydrolysis probes. These genes were selected to include a wide range of fold changes detected by microarray (e.g. 1.8-fold increase for Gli2 in DRG, versus over 100-fold increase for crystallin alpha B in DRG), as well as to include transcripts potentially affected by the extrinsic cue Sema3A (see below). The expression changes of all eight DRG genes selected for significant changes by microarray analysis were validated by qPCR (Table 1). Similarly, 4 out of 6 genes observed by microarray analysis to change significantly over time in SCG were validated by qPCR (Table 2). Figure 4 illustrates the time course of gene expression change in DRG (Figure 4A) and in SCG (Figure 4B) for those genes with significant change over time by both microarray and qPCR. Thus, we believe that many if not most of the changes identified by microarray analysis indeed reflect real changes in mRNA abundance in the two explant culture systems.

**Table 1:** Validation using quantitative real-time PCR (qPCR) of genes found to change significantly in DRG by microarray.

**Gene**	**Gene Title**	**Max. fold change by microarray^§^**	**Max. fold change by qPCR**	**p-value**
Cntn3	Contactin 3	2.6	11.0	0.007
Col18	Procollagen, type XVIII, alpha 1	4.3	8.1	0.0003
Cryab*	Crystallin, alpha B	123.0	42.5	<.0001
Fdps	Farnesyl diphosphate synthetase	2.4	2.0	<.0001
Gli2*	GLI-Kruppel family member GLI2	1.8	2.4	0.03**
Sparc	Secreted acidic cysteine rich glycoprotein	4.8	6.5	<.0001
Timp3*	Tissue inhibitor of metalloproteinase 3	2.6	3.6	0.003
Tubb3	Tubulin, beta 3	1.6	2.6	0.0002

p-value indicates significance of qPCR data for a quadratic model of change over time.

* Selection of these genes included probesets not part of the matched DRG-SCG dataset, as an additional screen for genes missed by these filters.

** Significance for this gene is for a linear model of change over time; this gene does not fit a quadratic model.

^§ ^Changes observed by microarray were statistically significant (quadratic model; p ≤ 0.05).

**Table 2:** Validation using quantitative real-time PCR (qPCR) of genes found to change significantly in SCG by microarray.

**Gene**	**Gene Title**	**Max. fold change by microarray^§^**	**Max. fold change by qPCR**	**p-value**
Col18	Procollagen, type XVIII, alpha 1	2.0	2.3	*0.59*
Fdps	Farnesyl diphosphate synthetase	1.8	2.3	0.05
Itga4	Integrin alpha 4	2.8	5.3	0.05**
L05*	RIKEN cDNA 1110035L05 gene	6.8	5.4	0.02
Ptprm	Receptor protein tyrosine phosphatase M	2.7	5.6	0.008
Timp3*	Tissue inhibitor of metalloproteinase 3	2.1	2.1	*0.25*

p-value indicates significance of qPCR data for a quadratic model of change over time. Italicized p-values for two genes indicate lack of statistical significance.

* Selection of these genes included probesets not part of the matched DRG-SCG dataset, as an additional screen for genes missed by these filters.

** Significance for this gene is for a linear model of change over time; this gene does not fit a quadratic model.

^§ ^Changes observed by microarray were statistically significant (quadratic model; p ≤ 0.05).

### Limited effects of Semaphorin 3A on transcription

Axon growth cones can exhibit dramatic changes in their morphologies, direction, and rate of growth in response to extrinsic protein cues encountered in the environment [62,63]. Semaphorin 3A (Sema3A) is one such diffusible axon guidance cue with potent repellent activity for both SCG and DRG neurons [63-65]. In addition, Sema3A has been demonstrated to affect local protein translation and axoplasmic transport, suggesting to us the possibility that Sema3A may interact with signaling cascades, including those that regulate gene transcription in the nucleus [27,66,67]. As an approach to determining whether exposure to an extrinsic cue such as Sema3A can elicit changes in gene expression, we cultured explants in the presence or absence of a heterologous source of Sema3A for varying amounts of time, and carried out microarray analyses as described above.

In spite of a robust effect by Sema3A on the morphology of neurite outgrowth in both culture systems (see Additional file 1 – Additional Figure 1), we found relatively few genes showing robust differences in gene expression in explants cultured in the presence or absence of Sema3A. As a quantitative measure, a gene was operationally defined as Sema3A-affected if it showed a 1.5 fold or greater difference between control and Sema3A-treated samples at any one time point, as well as a p-value ≤ 0.05 by quadratic analysis. Using these criteria, we observed 74 probesets affected by Sema3A in DRG, and 249 probesets affected by Sema3A in SCG (see Additional file 2 – Sema3A effects table). The magnitude of changes observed was subtle–for DRG probesets the median fold difference between control and Sema3A treated samples was 1.72 fold (IQR: 1.6, 1.72, 1.96; maximum 3.5), and for SCG probesets it was 1.76 fold (IQR: 1.6, 1.76, 2.04; maximum 4.68). With just one exception, however, none of the genes selected for validation by qPCR were shown to be regulated by Sema3A in either DRG or SCG (see below).

**Figure 5:**
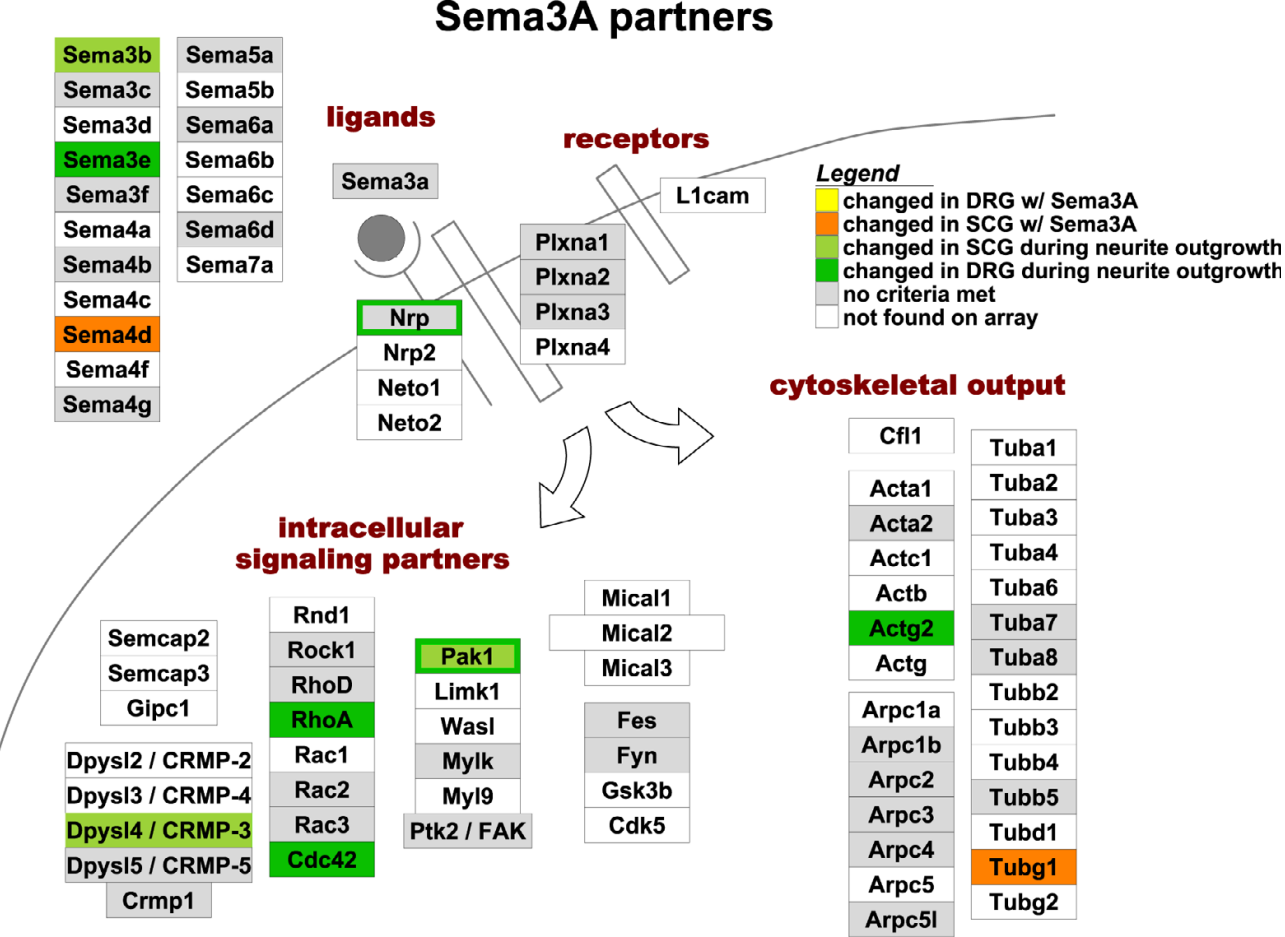
**Gene expression changes observed in Sema3A signaling pathway**. Model of Sema3A signaling pathway, colored by GenMAPP to depict under-representation of genes changed during Sema3A exposure, as compared to the number changed during neurite outgrowth *in vitro*. Criteria for changed genes are the same as for the microarray analysis. Genes on the MAPP include both validated interaction partners from the literature as well as their homologs. Gene names are listed in Additional file 2.

Taking these data at face value, it is difficult to confirm or rule out the possibility that Sema3A induces any meaningful changes at the transcriptional level. We therefore reasoned that if Sema3A causes a significant and biologically-relevant effect on transcription, the most likely genes affected might encode proteins either directly in the semaphorin signaling pathway or otherwise known to be involved in cell signaling or cytoskeletal dynamics. To test this idea, a candidate list was compiled of genes previously described to be downstream of Sema3A or related guidance cues [64,68-70]. GenMAPP was used to analyze this pathway for effects found during neurite outgrowth or exposure to Sema3A, and this revealed many more genes affected by neurite outgrowth than by Sema3A (Figure 5). This approach failed to reveal any significant enrichment of Sema3A-affected genes in this group relative to their representation in the whole dataset. The only two genes with potential Sema3A effects on this MAPP (Semaphorin 4D and Tubulin gamma 1) showed less than a 2-fold maximum difference between control and Sema3A-treated samples. We also analyzed a list of 360 guidance-related signaling molecules, including gene families and receptors for ephrins, netrins, transforming growth factor betas, neurotrophins, small GTP associated proteins, cadherins and other extracellular matrix molecules, protein kinases, and many other pathways [63,71-73]. Similar to Figure 5, GenMAPP analysis of this pathway revealed no significant enrichment of genes affected by Sema3A exposure (data not shown).

Quantitative PCR analysis was used to further investigate several potential Sema3A-affected transcripts found by microarray analysis. Measurement of a 2-fold change is near the detection limit of qPCR, so it is not surprising that for most genes analyzed, qPCR either did not reveal a substantial effect of Sema3A or else the effect was not statistically significant (Table 3 – DRG and Table 4 – SCG, see also Additional file 1 – Additional Figures 4 and 5). The only gene for which a statistically significant Sema3A effect was observed by qPCR–of a maximum 1.7 fold change–was tubulin beta 3 (Tubb3) in DRG (Table 3). Since Tubb3 is a cytoskeletal component, its upregulation could indicate an effect of extrinsic cue signaling on the neuronal cytoskeleton. The lack of validation for the majority of Sema3A-affected genes indicates that changes observed by microarray are either within the level of noise, or that very subtle effects are masked by technical and biological variability in these assays. However qPCR confirmed that there are no large-scale effects of Sema3A at the transcriptional level. Taken together, our results suggest that effects of the extrinsic cue Sema3A on neurite outgrowth in SCG and DRG are not manifested at the transcriptional level, but rather are focused on biochemical changes at the level of receptors and other signaling molecules localized to axonal or cytoplasmic compartments of the cell.

**Table 3:** Quantitative real-time PCR (qPCR) does not support Sema3A-induced changes in DRG observed by microarray.

**Gene**	**Gene Title**	**Max. difference by microarray^§^**	**Max. difference by qPCR**	**p-value**
Cntn3	Contactin 3	2.1	2.6	*0.34*
Col18	Procollagen, type XVIII, alpha 1	1.6	2.5	*0.38*
Fdps	Farnesyl diphosphate synthetase	1.7	1.4	*0.18*
Gli2*	GLI-Kruppel family member GLI2	1.5	1.8	*0.66*
Tubb3	Tubulin, beta 3	1.3^†^	1.7	0.01

p-value indicates significance of qPCR data for a quadratic model of change over time. Italicized p-values indicate lack of statistical significance.

* Selection of these genes included probesets not part of the matched DRG-SCG dataset, as an additional screen for genes missed by these filters.

^§ ^Mock-Sema3A differences observed by microarray were statistically significant (quadratic model; p ≤ 0.05).

^† ^By microarray analysis, the mock-Sema3A differences observed for Tubb3 were not statistically significant.

**Table 4:** Quantitative real-time PCR (qPCR) does not support Sema3A-induced changes in SCG observed by microarray.

**Gene**	**Gene Title**	**Max. difference by microarray^§^**	**Max. difference by qPCR**	**p-value**
Col18	Procollagen, type XVIII, alpha 1	2.7	2.5	*0.66*
Cryab*	Crystallin, alpha B	2.5	2.2	*0.21*
Fdps	Farnesyl diphosphate synthetase	2.0	2.3	*0.22*
L05*	RIKEN cDNA 1110035L05 gene	4.3	1.5	*0.82*
Lmo4*	LIM domain only 4	2.1	1.6	*0.26*
Ptprm	Receptor protein tyrosine phosphatase M	2.7	2.8	*0.27*
Rce1	Ras and a-factor-converting enzyme 1 homolog	1.4	1.3	*0.77*
Timp3*	Tissue inhibitor of metalloproteinase 3	1.7	2.6	*0.18*
Unc119	unc-119 homolog	1.8	1.3	*0.95*
Uty*	Ubiquitously transcribed gene, Y chromosome	3.4	2.6	*0.33*

Italicized p-values indicates lack of statistical significance of qPCR data for a quadratic model of change over time.

* Selection of these genes included probesets not part of the matched DRG-SCG dataset, as an additional screen for genes missed by these filters.

^§ ^Mock-Sema3A differences observed by microarray were statistically significant (quadratic model; p ≤ 0.05).

## Discussion

### Comparing across *in vitro *models

We assessed the gene expression patterns of neurite outgrowth for two different neuronal populations over a wide range of time points. This comparative approach is advantageous because it reveals commonalities across divergent cell types of the peripheral nervous system. As expected for tissues serving different biological functions and isolated from different chronological ages, there are differences in gene expression between these two explant types. However of the genes that are actively engaged by neurite outgrowth in both SCG and DRG, there are more similarities in direction of change than divergences (see Figure 1 for illustration). These commonalities in gene expression are likely to be related to the similar activity undergone by these explants over time. The observation that so many genes are changing similarly in both neuronal tissue types suggests that these effects may be relevant to other neurons as well. The cluster of transcriptional regulators highlighted in this analysis demonstrates one such group of genes for future investigation.

### Sema3A does not elicit significant transcriptional changes

Previous data on the growth-cone collapsing effects of Sema3A, as well as its ability to affect axoplasmic transport, suggested to us that Sema3A could act at multiple levels, with local effects on growth cone morphology as well as downstream effects on the nucleus [27,28,63-67]. Other developmental guidance cues such as bone morphogenetic proteins, Sonic hedgehog, and wingless-related proteins have both transcriptional and local effects [71,74-86]. To address whether the gene expression patterns of neurite outgrowth or regeneration were affected by extrinsic factors such as Sema3A, we conducted parallel experiments with and without Sema3A in the culture environment. For this extrinsic cue, minimal changes in the number and magnitude of gene expression were observed, despite careful scrutiny of both individual genes and a systematic search for pathway-level effects. We were unable to experimentally validate any of the Sema3A-related changes observed by microarray. Although we cannot rule out the possibility that we missed other effects due to experimental limitations (e.g. changes in a gene not on our microarrays, at another time point, or below the level of detection), our results nonetheless suggest that the inhibitory effects of Sema3A at sites of injury and regeneration *in vivo *are mediated locally and not via transcriptional changes in the responding cells.

### Parallels and insights for *in vivo *regeneration

To discern potentially important candidates within the genes commonly affected during outgrowth by SCG and DRG, we compared our data to several previously published studies of neurite regeneration *in vivo*. This analysis revealed commonalities between neurite outgrowth *in vitro *and regeneration *in vivo*, and it also revealed differences between those two. The genes affected in both cases are logically the ones that are most likely to be involved in both processes. Among the differences, it is expected that some transcripts expressed and affected in adult models of regeneration are particular to mature neurons with functional synaptic connections, and that others are due to interactions with the inflammatory and inhibitory extracellular environment after injury. Our data facilitates the interpretation of *in vivo *data because it provides an opportunity to focus on genes involved in growth competence, in the absence of reactions to loss of synaptic input and to the extracellular environment of injury.

The differences observed between gene expression patterns of *in vivo *regeneration and *in vitro *outgrowth fall into two main categories. First, there are genes that are present on the array platforms used, but that have a low intensity signal that does not appear to change over time. Examples of this group included galanin, GFAP, and receptors for benzodiazepine, serotonin, or glutamate. Interpretation of this group is difficult, because a failure to observe a strong signal may indicate either poor signal detection by the array probes, or may indicate meaningful transcript absence or repression in these cultures.

A second category of differences between *in vivo *regeneration and *in vitro *outgrowth yields more insights. In this group of regeneration-associated genes, the transcripts are observed to change during *in vitro *outgrowth, but the directionality of change is different. For instance, genes that are commonly upregulated in regeneration models but that are down-regulated during *in vitro *outgrowth include arginase 1, Npy, and Gadd45A [10,19,21-23,55,56]. The observation that these transcripts change over time in our cultures indicates that their expression is being regulated in these models, and it also suggests that their upregulation (as observed after adult injury *in vivo*) is not required for neurite regrowth by DRG or SCG neurons *in vitro*. Since our models use an extrinsically controlled *in vitro *environment, these observations suggest that the changes observed in these transcripts after injury *in vivo *may have more to do with responses to inhibitory aspects of the *in vivo *environment or to interactions with other cell types in the regenerating areas, rather than with neurite outgrowth.

Finally, the process of extending neurites, which is common to both regeneration *in vivo *and outgrowth *in vitro*, suggests that there may be commonalities in the gene expression programs involved. This was confirmed in part by our meta-analysis comparison of several regeneration models *in vivo *to the genes changed during outgrowth *in vitro*. Similar up- or down-regulation was observed for genes including lysozyme, secretogranin 2, and the transcription factors 1sl1 and Atf3. Additional commonalities were revealed by a comparison of the genes most highly expressed in both explant models (Figure 2B, see also Additional file 2). As described earlier, embryonic neurons are already growth competent in their gene expression patterns, and thus may already express certain regeneration-associated genes at the time of dissection. A number of the genes most highly expressed in both explant types, such as GAP43, CAP23/BASP1, and SCG10, as well as others listed above, are well documented for their roles in regeneration [57-61]. This group of genes that are highly expressed and maintained in these embryonic explants may thus be a window into potential regeneration-associated genes. These candidates may be ideal for further investigation as regeneration-promoting genes, for instance by testing their ability to stimulate neurite outgrowth of adult neurons as was demonstrated for GAP43 and CAP23/BASP1 [8,9].

## Conclusion

By investigating intrinsic transcriptional activity in DRG and SCG as two distinct *in vitro *models of neurite outgrowth, we have demonstrated commonalities in gene expression relevant to this process. In addition, through a meta-analysis comparison of these results to several large-scale studies of gene expression during regeneration *in vivo*, we have discerned that many transcripts affected *in vivo *are also highly expressed or changed during outgrowth *in vitro*. Together these results demonstrate the usefulness of our *in vitro *models in identifying programs of gene expression intrinsic to regenerating neurons. We also found that despite its potent effects as an inhibitory extrinsic cue and potential for retrograde signaling, Sema3A does not appear to significantly affect the intrinsic transcriptional activities involved in neurite outgrowth. Thus, the robust effects of semaphorin signaling on axon growth cone guidance are most likely restricted to local mechanisms not involving changes in gene expression in the nucleus. Our analyses present several groups of genes relevant for future testing of potential roles in neurite outgrowth or regeneration.

## Methods

### Cell culture

For each culture, embryonic explants were dissected from two litters of outbred CD1 mice of either embryonic day 13 (E13) for SCG or E12 for DRG. Collagen drop cultures were as per published methods [87,88], using either commercial rat tail collagen (BD Biosciences, San Jose, CA) or rat tail collagen prepared as per Guthrie and Lumsden [89]. After dissection, two to four explants were embedded in a collagen sandwich with an aggregate of Cos7 cells. Culture medium for SCG consisted of D-MEM/F-12 medium with 100 U/ml penicillin/streptomycin (P/S), 2 mM L-Glutamine (all Invitrogen, Carlsbad, CA), 1 mg/ml bovine albumin, and 25 ng/ml of NGF-7S (both Sigma, St. Louis, MO). Culture medium for DRG consisted of 1:1 F-12 and Opti-MEM I, 0.5% heat-inactivated horse serum, 2 mM GlutaMAX, 100 U/ml P/S, 40 mM glucose, and 25 ng/ml NGF.

The Cos7 cells were either mock-transfected or transiently transfected with 5 μg Sema3A plasmid [90], using Lipofectamine 2000 (Invitrogen) as per manufacturer's instructions. Cells were aggregated by hanging drop [91]. Semaphorin expression was confirmed by observation of outgrowth and axon repulsion in a parallel explant culture for each transfection.

### RNA isolation & amplification

A reference pool for microarray comparisons was created by dissecting explants as above, and transferring tissues to Trizol RNA isolation reagent (Invitrogen) without culturing. For SCG, 42 ganglia (from four litters) were collected into four samples. For DRG, 94 ganglia (from three litters) were collected into six samples. RNA was isolated from these samples and quantitated as below.

Cultured DRG or SCG were harvested into Trizol at 2, 5, 12, 24, 40, and 65 hours (final time point for SCG only). RNA was isolated as per Invitrogen protocol, treated with RNase-free DNase (Promega, Madison, WI), cleaned via Zymo-RNA column (ZYMO Research, Orange, CA), and quantitated by Ribogreen assay (Molecular Probes/Invitrogen). For DRG samples, all five time points of each replicate were collected consecutively from one culture setup, making them continuous replicates. SCG sample tissue was more limited, and therefore the microarray replicates are discontinuous, composed of intermingled time points from multiple culture setups. For the Taqman analysis where it was feasible to use less RNA, two new SCG replicates were collected as consecutive replicates. Two biological replicates for each tissue type were collected for hybridization to Affymetrix arrays. One of these and two additional biological replicates were used for quantitative RT-PCR analysis.

For each microarray sample, 50–500 ng of total RNA was amplified using a modified Eberwine procedure [92,93]. 200 ng of T7-oligo dT primer was added to each (AAATTAATACGACTCACTATAGGGAGACCACA(T)21)). A 20 μl reverse transcription reaction was carried out for each sample using Superscript III (SSIII; Invitrogen) as per manufacturer's protocol, with reaction time extended to 16 hours. Second strand cDNA synthesis utilized DNA ligase, DNA polymerase I holoenzyme (both New England Biolabs, Ipswich, MA), dNTPs, and second strand buffer (Invitrogen) for 2–6 hours at 16 degrees C. cDNA was isolated by phenol chloroform extraction and precipitated. cRNA was produced from cDNA with an Ambion T7 Megascript kit, and collected via Qiagen RNeasy column. RNA was quantitated by spectrophotometer and gel electrophoresis. This cRNA was used directly for cDNA labeling for spotted microarrays (see Additional file 1), or processed through a second round amplification for Affymetrix arrays.

Second round amplification followed the Affymetrix Small-Sample Target Labeling Assay Version II from step 6 onward. Biotin-labeled cRNA was made using the BioArray High Yield RNA Transcript Labeling kit (Enzo Diagnostics, Farmingdale, NY) and eluted by RNeasy column. cRNA was fragmented according to Affymetrix protocol. All SCG samples were hybridized to Affymetrix MG-U74v2 A and B arrays, and DRG samples were hybridized to MOE430A arrays. All hybridizations were performed via Affymetrix protocols at the Stanford Protein and Nucleic Acid Biotechnology Facility.

### Image analysis and normalization

Affymetrix array hybridization files (.cel, .exp, and .dat) were uploaded to Genetraffic Uno (Iobion, La Jolla, CA). Since SCG and DRG samples used two different series of Affymetrix arrays, the data for each was extracted separately. gcrma (version 1.1) was used to extract data [94-96]. To balance the median array intensity between array platforms, a constant value of 1.83 was added to the logged intensity values for the SCG (MG-U74v2) arrays. All files were quality assessed with the Affy and AffyExtensions packages for Bioconductor in R [97], and no chips were excluded for quality reasons. Based on Affymetrix probeset descriptions and the disproportionate number of low-intensity probesets in these categories, probesets annotated as incomplete (i), rules dropped (r), or cross-hybridizing (x) were excluded. Data for each group of arrays was baselined to the appropriate (SCG or DRG) acutely-dissected reference pool. This facilitated comparison between Affymetrix platforms as well as to spotted microarray data.

The Affymetrix same-species best-match comparison spreadsheet was used to match probe sets between platforms used for SCG and DRG hybridizations. Due to redundancies in the older array design, multiple probe sets on the U74v2 arrays have the same best match in the 430A design. For these cases, the Affymetrix probe descriptions were used to determine a single best match, by selecting if possible the probeset match without any annotation qualifier, followed in decreasing preference those with g, f, or s annotations. The final dataset included 11,268 probesets matched across platforms; (see Additional file 3 these were used for all further analysis.

### Statistical analysis

gcrma-normalized data from SCG and DRG were analyzed separately to determine if changes in gene expression over time were statistically significant. The limma package in R was used to test if changes in gene expression over time fit a quadratic or linear model [98]. Data was used for clustering and further analysis if the p value of a moderated F statistic for either model was ≤ 0.05, and the maximum fold change over time (between any two time points) was at least 1.5 [99]. A false discovery rate (FDR) test was run as well, but results of the FDR were not used in filtering data for clustering or further analysis. To analyze effects of Sema3A, a quadratic model testing for the interaction of time and treatment was used. For neurite outgrowth, a one-arm model was used. For analysis of Sema3A effects, a two-arm model was used to include the effect of treatment. In the DRG data, a replicate variable was also included, because the samples were biologically continuous. Regression analysis, including both quadratic and linear effects, was used to test the significance of changes over time and with Sema3A treatment in the qPCR data, after data extraction as described below.

### Clustering, annotation, and visualization

Microarray data were clustered using the clustering algorithm Hierarchical Ordered Partitioning and Collapsing Hybrid (HOPACH) [29], using the cosine angle metric (uncentered correlation). Clusters were visualized in Java Treeview 1.0.4 after sorting by the HOPACH final order. Visualization of the time course of expression for individual genes was done using a plotting function in R.

To annotate the biological functions of HOPACH clusters, we used Gene Map Annotator and Pathway Profiler (GenMAPP) [31,32]. For this purpose, the main order clusters (called sub-clusters here) provided by HOPACH were used, rather than the final-order clusters that are essentially unique for every gene. Each HOPACH cluster list was used to create a gene expression database in GenMAPP, with first or second level HOPACH cluster numbers as filter criteria (see for example Additional file 4). MAPPFinder was used to search for over-representation of Gene Ontology (GO) terms [33,34] in each cluster. Significant biological associations were indicated if three or more genes in a given GO term were changed, with a permuted p value ≤ 0.05. Instances where the adjusted p value (adjusted for multiple hypothesis testing) were also less than 0.05 are noted.

### Matching to published data on in vivo regeneration models

From Costigan et al. we extracted 187 unique genes significantly regulated at least 1.5 fold in DRG, at three days after sciatic nerve axotomy [21]. Changes were analyzed in L4 and L5 (lumbar) DRG on the side ipsilateral to the injury, and compared to uninjured DRG from the opposite side. Hybridization was done using Affymetrix Rat U34A arrays.

From Xiao et al. we extracted 88 unique genes significantly regulated at least 2 fold in DRG, at two days after sciatic nerve axotomy [23]. Changes were analyzed in L4 and L5 DRG of lesioned animals versus non-lesioned animals. Hybridization was done using cDNA array membranes of their own production, as well as CLONTECH Atlas arrays.

From Boeshore et al. we extracted 248 unique genes significantly regulated at least 2 fold in SCG, at two days after axotomy of the internal and external carotid nerves [19]. Changes were analyzed in SCGs of lesioned animals versus sham-operated animals. Hybridization was done using Affymetrix Rat U34A arrays.

Accession numbers for Xiao et al. were annotated using Stanford SOURCE to provide an Entrez Gene ID [100]. Entrez Gene IDs for the other studies were extracted from Affymetrix Rat U34A annotations. Duplicate probeset-Entrez Gene matches and probesets with no Entrez Gene ID were excluded. Mouse Entrez Gene IDs for our data came from Affymetrix MOE430 annotation. NCBI's Homologene database was used to match rat and mouse orthologs. A Matlab script by Jeremy Heil was used for proportional Venn diagrams.

### Real-time RT-PCR validation

For each gene of interest, sequence data was compared from NCBI's Unigene, Entrez Gene (formerly Locuslink) resources, and Ensembl [101,102]. Whenever possible, primer-probe pairs were designed to span an intron of 1500 or more base pairs. Primers and Taqman (Applied Biosystems, Foster City, CA) probes were selected using Primer Express 2.0.0 (Applied Biosystems) and DSGene 1.5 (Accelrys, San Diego, CA). The average amplicon size was 100 base pairs. Primer and probe sequences are listed in Additional file 2, along with the NCBI Refseq for each gene.

Unamplified total RNA was used for quantitative real-time RT-PCR (qPCR) validation, as per Applied Biosystems protocols. Two of the three biological replicates were independent of those used for the Affymetrix arrays. Briefly, 50 ng of total RNA was primed with random hexamers and reverse transcribed with SSIII as above. Each reaction was aliquotted and a 1/20 dilution used as a template for qPCR. qPCR used Platinum Taq (Invitrogen) with recommended buffer and dNTP concentrations, and were run in an MJ Opticon Monitor.

Expression was normalized by the comparative threshold cycle method [103] using one of two reference genes, Stathmin 2 (Stmn2/SCG10) or WD repeat domain 4 (Wdr4). For each sample, the threshold cycle of detection was normalized to a parallel loading control of the reference gene. This was then calibrated to the 2 hour time point, which was set to zero. All time points for a given replicate were handled in parallel.

#### Data accessibility

The gene expression data described here are accessible through GEO Series accession number GSE9744.

## Authors' contributions

MLS contributed to conception of the study, conducted all experiments, and drafted the manuscript. KV participated in design of the study and performed the statistical analyses shown here. YCT and TPS provided additional statistical analyses and input on the design of the study. CG and JN contributed to the conception of the study and participated in its design and execution. All authors contributed to and approved the final manuscript.

## Supplementary Material

Additional file 1 **Additional Figures and Additional Methods**. Contains Additional Figures 1–5, figure legends and Additional Methods. Click here for file

Additional file 2 **Abbreviations**. Lists the abbreviations and corresponding gene names used in the Sema3A pathway of Figure 4 and in the annotation of regeneration-associated genes (shown on Figure 1 and in Additional file 1). **Meta-analysis**. Lists the genes extracted from the meta-analysis of our data with three published studies of genes changed during *in vivo *neurite regeneration. The table indicates where matching probesets were found in the present data, as well as if those genes were significantly changed in the present study. **Top 5% intersection**. Lists the top 5%, 2% and 1% intersection of genes with the highest intensity, or expression level, in both DRG and SCG. The table includes the median intensity for each gene in DRG and SCG, as well as the Affymetrix probeset for each gene. **Q-RT-PCR**. Lists the genes used for qRT-PCR analysis, the main reference sequence from NCBI used to design each set of primers, and the sequences of each forward, probe, and reverse primer. The table also lists the Affymetrix probesets matching each of these genes. **Sema3A effects**. Lists the genes observed by microarray to be significantly affected by exposure to Sema3A. The table includes the maximum fold difference after Sema3A exposure, as well as the quadratic analysis p-value for significance of this difference. Click here for file

Additional file 3 **DRG-SCG dataset**. Lists all 11,268 probe sets found in the DRG-SCG dataset, along with relevant annotation and microarray data. Statistics and measures of maximum change over time are listed for all genes. This list contains the data and criteria used to generate HOPACH clusters and GenMAPP filters. Click here for file

Additional file 4 **DRG SCG gMAPP.gex**. GenMAPP expression database of the 712 genes jointly affected during neurite outgrowth by DRG and SCG. Includes HOPACH cluster levels and other criteria for analyzing and visualizing affected genes and pathways in GenMAPP. GenMAPP software is available online [104]. Click here for file

## References

[B1] Bulsara KR, Iskandar BJ, Villavicencio AT, Skene JH (2002). A new millenium for spinal cord regeneration: growth-associated genes. Spine.

[B2] Smith DS, Skene JH (1997). A transcription-dependent switch controls competence of adult neurons for distinct modes of axon growth. J Neurosci.

[B3] Teng FY, Tang BL (2006). Axonal regeneration in adult CNS neurons--signaling molecules and pathways. J Neurochem.

[B4] Richardson PM, Issa VM (1984). Peripheral injury enhances central regeneration of primary sensory neurones. Nature.

[B5] Neumann S, Woolf CJ (1999). Regeneration of dorsal column fibers into and beyond the lesion site following adult spinal cord injury. Neuron.

[B6] Seijffers R, Allchorne AJ, Woolf CJ (2006). The transcription factor ATF-3 promotes neurite outgrowth. Mol Cell Neurosci.

[B7] Seijffers R, Mills CD, Woolf CJ (2007). ATF3 increases the intrinsic growth state of DRG neurons to enhance peripheral nerve regeneration. J Neurosci.

[B8] Buffo A, Holtmaat AJ, Savio T, Verbeek JS, Oberdick J, Oestreicher AB, Gispen WH, Verhaagen J, Rossi F, Strata P (1997). Targeted overexpression of the neurite growth-associated protein B-50/GAP-43 in cerebellar Purkinje cells induces sprouting after axotomy but not axon regeneration into growth-permissive transplants. J Neurosci.

[B9] Bomze HM, Bulsara KR, Iskandar BJ, Caroni P, Skene JH (2001). Spinal axon regeneration evoked by replacing two growth cone proteins in adult neurons. Nat Neurosci.

[B10] Bonilla IE, Tanabe K, Strittmatter SM (2002). Small proline-rich repeat protein 1A is expressed by axotomized neurons and promotes axonal outgrowth. J Neurosci.

[B11] Caroni P (1998). Neuro-regeneration: plasticity for repair and adaptation. Essays Biochem.

[B12] Filbin MT (2003). Myelin-associated inhibitors of axonal regeneration in the adult mammalian CNS. Nat Rev Neurosci.

[B13] Pasterkamp RJ, Verhaagen J (2001). Emerging roles for semaphorins in neural regeneration. Brain Res Brain Res Rev.

[B14] Ng CE, Tang BL (2002). Nogos and the Nogo-66 receptor: factors inhibiting CNS neuron regeneration. J Neurosci Res.

[B15] Yiu G, He Z (2003). Signaling mechanisms of the myelin inhibitors of axon regeneration. Curr Opin Neurobiol.

[B16] Barnett SC, Riddell JS (2004). Olfactory ensheathing cells (OECs) and the treatment of CNS injury: advantages and possible caveats. J Anat.

[B17] Schwab ME (2002). Repairing the injured spinal cord. Science.

[B18] Chong MS, Woolf CJ, Turmaine M, Emson PC, Anderson PN (1996). Intrinsic versus extrinsic factors in determining the regeneration of the central processes of rat dorsal root ganglion neurons: the influence of a peripheral nerve graft. J Comp Neurol.

[B19] Boeshore KL, Schreiber RC, Vaccariello SA, Sachs HH, Salazar R, Lee J, Ratan RR, Leahy P, Zigmond RE (2004). Novel changes in gene expression following axotomy of a sympathetic ganglion: a microarray analysis. J Neurobiol.

[B20] Schmitt AB, Breuer S, Liman J, Buss A, Schlangen C, Pech K, Hol EM, Brook GA, Noth J, Schwaiger FW (2003). Identification of regeneration-associated genes after central and peripheral nerve injury in the adult rat. BMC Neurosci.

[B21] Costigan M, Befort K, Karchewski L, Griffin RS, D'Urso D, Allchorne A, Sitarski J, Mannion JW, Pratt RE, Woolf CJ (2002). Replicate high-density rat genome oligonucleotide microarrays reveal hundreds of regulated genes in the dorsal root ganglion after peripheral nerve injury. BMC Neurosci.

[B22] Tanabe K, Bonilla I, Winkles JA, Strittmatter SM (2003). Fibroblast Growth Factor-Inducible-14 Is Induced in Axotomized Neurons and Promotes Neurite Outgrowth. J Neurosci.

[B23] Xiao HS, Huang QH, Zhang FX, Bao L, Lu YJ, Guo C, Yang L, Huang WJ, Fu G, Xu SH, Cheng XP, Yan Q, Zhu ZD, Zhang X, Chen Z, Han ZG (2002). Identification of gene expression profile of dorsal root ganglion in the rat peripheral axotomy model of neuropathic pain. Proc Natl Acad Sci U S A.

[B24] De Biase A, Knoblach SM, Di Giovanni S, Fan C, Molon A, Hoffman EP, Faden AI (2005). Gene expression profiling of experimental traumatic spinal cord injury as a function of distance from impact site and injury severity. Physiol Genomics.

[B25] Hu J, Fink D, Mata M (2002). Microarray analysis suggests the involvement of proteasomes, lysosomes, and matrix metalloproteinases in the response of motor neurons to root avulsion. Eur J Neurosci.

[B26] Fan M, Mi R, Yew DT, Chan WY (2001). Analysis of gene expression following sciatic nerve crush and spinal cord hemisection in the mouse by microarray expression profiling. Cell Mol Neurobiol.

[B27] Li C, Sasaki Y, Takei K, Yamamoto H, Shouji M, Sugiyama Y, Kawakami T, Nakamura F, Yagi T, Ohshima T, Goshima Y (2004). Correlation between semaphorin3A-induced facilitation of axonal transport and local activation of a translation initiation factor eukaryotic translation initiation factor 4E. J Neurosci.

[B28] Togashi H, Schmidt EF, Strittmatter SM (2006). RanBPM contributes to Semaphorin3A signaling through plexin-A receptors. J Neurosci.

[B29] van der Laan MJ, Pollard KS, Bryan J (2003). A New Partitioning Around Medoids Algorithm. Journal of Statistical Computation and Simulation.

[B30] Eisen MB, Spellman PT, Brown PO, Botstein D (1998). Cluster analysis and display of genome-wide expression patterns. PNAS.

[B31] Dahlquist KD, Salomonis N, Vranizan K, Lawlor SC, Conklin BR (2002). GenMAPP, a new tool for viewing and analyzing microarray data on biological pathways. Nat Genet.

[B32] Doniger SW, Salomonis N, Dahlquist KD, Vranizan K, Lawlor SC, Conklin BR (2003). MAPPFinder: using Gene Ontology and GenMAPP to create a global gene-expression profile from microarray data. Genome Biol.

[B33] Harris MA, Clark J, Ireland A, Lomax J, Ashburner M, Foulger R, Eilbeck K, Lewis S, Marshall B, Mungall C, Richter J, Rubin GM, Blake JA, Bult C, Dolan M, Drabkin H, Eppig JT, Hill DP, Ni L, Ringwald M, Balakrishnan R, Cherry JM, Christie KR, Costanzo MC, Dwight SS, Engel S, Fisk DG, Hirschman JE, Hong EL, Nash RS, Sethuraman A, Theesfeld CL, Botstein D, Dolinski K, Feierbach B, Berardini T, Mundodi S, Rhee SY, Apweiler R, Barrell D, Camon E, Dimmer E, Lee V, Chisholm R, Gaudet P, Kibbe W, Kishore R, Schwarz EM, Sternberg P, Gwinn M, Hannick L, Wortman J, Berriman M, Wood V, de la Cruz N, Tonellato P, Jaiswal P, Seigfried T, White R, Gene Ontology Consortium (2004). The Gene Ontology (GO) database and informatics resource. Nucleic Acids Res.

[B34] (2001). Creating the gene ontology resource: design and implementation. Genome Res.

[B35] Raivich G, Makwana M (2007). The making of successful axonal regeneration: genes, molecules and signal transduction pathways. Brain Res Rev.

[B36] Goldberg JL (2003). How does an axon grow?. Genes Dev.

[B37] Middleton G, Davies AM (2001). Populations of NGF-dependent neurones differ in their requirement for BAX to undergo apoptosis in the absence of NGF/TrkA signalling in vivo. Development.

[B38] White FA, Keller-Peck CR, Knudson CM, Korsmeyer SJ, Snider WD (1998). Widespread elimination of naturally occurring neuronal death in Bax-deficient mice. J Neurosci.

[B39] Snider WD (1994). Functions of the neurotrophins during nervous system development: what the knockouts are teaching us. Cell.

[B40] Snider WD, Wright DE (1996). Neurotrophins cause a new sensation. Neuron.

[B41] Francis NJ, Landis SC (1999). Cellular and molecular determinants of sympathetic neuron development. Annu Rev Neurosci.

[B42] Huber K, Kuehnel F, Wyatt S, Davies AM (2000). TrkB expression and early sensory neuron survival are independent of endogenous BDNF. J Neurosci Res.

[B43] Lindwall C, Kanje M (2005). The Janus role of c-Jun: cell death versus survival and regeneration of neonatal sympathetic and sensory neurons. Exp Neurol.

[B44] Knapska E, Kaczmarek L (2004). A gene for neuronal plasticity in the mammalian brain: Zif268/Egr-1/NGFI-A/Krox-24/TIS8/ZENK?. Prog Neurobiol.

[B45] Robinson GA (1994). Immediate early gene expression in axotomized and regenerating retinal ganglion cells of the adult rat. Brain Res Mol Brain Res.

[B46] Amit I, Citri A, Shay T, Lu Y, Katz M, Zhang F, Tarcic G, Siwak D, Lahad J, Jacob-Hirsch J, Amariglio N, Vaisman N, Segal E, Rechavi G, Alon U, Mills GB, Domany E, Yarden Y (2007). A module of negative feedback regulators defines growth factor signaling. Nat Genet.

[B47] Campbell DB, Levitt P (2003). Regionally restricted expression of the transcription factor c-myc intron 1 binding protein during brain development. J Comp Neurol.

[B48] Rubin E (1985). Development of the rat superior cervical ganglion: ganglion cell maturation. J Neurosci.

[B49] Enomoto H, Crawford PA, Gorodinsky A, Heuckeroth RO, Johnson EM, Milbrandt J (2001). RET signaling is essential for migration, axonal growth and axon guidance of developing sympathetic neurons. Development.

[B50] Mirnics K, Koerber HR (1995). Prenatal development of rat primary afferent fibers: I. Peripheral projections. J Comp Neurol.

[B51] Mirnics K, Koerber HR (1995). Prenatal development of rat primary afferent fibers: II. Central projections. J Comp Neurol.

[B52] Ozaki S, Snider WD (1997). Initial trajectories of sensory axons toward laminar targets in the developing mouse spinal cord. J Comp Neurol.

[B53] Wang H, Sun H, Della Penna K, Benz RJ, Xu J, Gerhold DL, Holder DJ, Koblan KS (2002). Chronic neuropathic pain is accompanied by global changes in gene expression and shares pathobiology with neurodegenerative diseases. Neuroscience.

[B54] Valder CR, Liu JJ, Song YH, Luo ZD (2003). Coupling gene chip analyses and rat genetic variances in identifying potential target genes that may contribute to neuropathic allodynia development. J Neurochem.

[B55] Befort K, Karchewski L, Lanoue C, Woolf CJ (2003). Selective up-regulation of the growth arrest DNA damage-inducible gene Gadd45 alpha in sensory and motor neurons after peripheral nerve injury. Eur J Neurosci.

[B56] Landry M, Holmberg K, Zhang X, Hokfelt T (2000). Effect of axotomy on expression of NPY, galanin, and NPY Y1 and Y2 receptors in dorsal root ganglia and the superior cervical ganglion studied with double-labeling in situ hybridization and immunohistochemistry. Exp Neurol.

[B57] Mason MR, Lieberman AR, Grenningloh G, Anderson PN (2002). Transcriptional upregulation of SCG10 and CAP-23 is correlated with regeneration of the axons of peripheral and central neurons in vivo. Mol Cell Neurosci.

[B58] Skene JH (1989). Axonal growth-associated proteins. Annu Rev Neurosci.

[B59] Van der Zee CE, Nielander HB, Vos JP, Lopes da Silva S, Verhaagen J, Oestreicher AB, Schrama LH, Schotman P, Gispen WH (1989). Expression of growth-associated protein B-50 (GAP43) in dorsal root ganglia and sciatic nerve during regenerative sprouting. J Neurosci.

[B60] Mason MR, Lieberman AR, Anderson PN (2003). Corticospinal neurons up-regulate a range of growth-associated genes following intracortical, but not spinal, axotomy. Eur J Neurosci.

[B61] Pellier-Monnin V, Astic L, Bichet S, Riederer BM, Grenningloh G (2001). Expression of SCG10 and stathmin proteins in the rat olfactory system during development and axonal regeneration. J Comp Neurol.

[B62] Tessier-Lavigne M, Goodman CS (1996). The molecular biology of axon guidance. Science.

[B63] Yu TW, Bargmann CI (2001). Dynamic regulation of axon guidance. Nat Neurosci.

[B64] Skaper SD, Moore SE, Walsh FS (2001). Cell signalling cascades regulating neuronal growth-promoting and inhibitory cues. Prog Neurobiol.

[B65] Fiore R, Puschel AW (2003). The function of semaphorins during nervous system development. Front Biosci.

[B66] Goshima Y, Hori H, Sasaki Y, Yang T, Kagoshima-Maezono M, Li C, Takenaka T, Nakamura F, Takahashi T, Strittmatter SM, Misu Y, Kawakami T (1999). Growth cone neuropilin-1 mediates collapsin-1/Sema III facilitation of antero- and retrograde axoplasmic transport. J Neurobiol.

[B67] Goshima Y, Kawakami T, Hori H, Sugiyama Y, Takasawa S, Hashimoto Y, Kagoshima-Maezono M, Takenaka T, Misu Y, Strittmatter SM (1997). A novel action of collapsin: collapsin-1 increases antero- and retrograde axoplasmic transport independently of growth cone collapse. J Neurobiol.

[B68] Liu BP, Strittmatter SM (2001). Semaphorin-mediated axonal guidance via Rho-related G proteins. Curr Opin Cell Biol.

[B69] He Z, Wang KC, Koprivica V, Ming G, Song HJ (2002). Knowing how to navigate: mechanisms of semaphorin signaling in the nervous system. Sci STKE.

[B70] Pasterkamp RJ, Kolodkin AL (2003). Semaphorin junction: making tracks toward neural connectivity. Curr Opin Neurobiol.

[B71] Schnorrer F, Dickson BJ (2004). Axon guidance: morphogens show the way. Curr Biol.

[B72] Grunwald IC, Klein R (2002). Axon guidance: receptor complexes and signaling mechanisms. Curr Opin Neurobiol.

[B73] Chisholm A, Tessier-Lavigne M (1999). Conservation and divergence of axon guidance mechanisms. Curr Opin Neurobiol.

[B74] Li X, Cao X (2003). BMP signaling and HOX transcription factors in limb development. Front Biosci.

[B75] Packard M, Mathew D, Budnik V (2003). Wnts and TGF beta in synaptogenesis: old friends signalling at new places. Nat Rev Neurosci.

[B76] Kronenberg HM (2003). Developmental regulation of the growth plate. Nature.

[B77] Dale TC (1998). Signal transduction by the Wnt family of ligands. Biochem J.

[B78] Marti E, Bovolenta P (2002). Sonic hedgehog in CNS development: one signal, multiple outputs. Trends Neurosci.

[B79] Butler SJ, Dodd J (2003). A role for BMP heterodimers in roof plate-mediated repulsion of commissural axons. Neuron.

[B80] Dionne MS, Brunet LJ, Eimon PM, Harland RM (2002). Noggin is required for correct guidance of dorsal root ganglion axons. Dev Biol.

[B81] Salinas PC (2003). Synaptogenesis: Wnt and TGF-beta take centre stage. Curr Biol.

[B82] Hall AC, Lucas FR, Salinas PC (2000). Axonal remodeling and synaptic differentiation in the cerebellum is regulated by WNT-7a signaling. Cell.

[B83] Yoshikawa S, McKinnon RD, Kokel M, Thomas JB (2003). Wnt-mediated axon guidance via the Drosophila Derailed receptor. Nature.

[B84] Lyuksyutova AI, Lu CC, Milanesio N, King LA, Guo N, Wang Y, Nathans J, Tessier-Lavigne M, Zou Y (2003). Anterior-posterior guidance of commissural axons by Wnt-frizzled signaling. Science.

[B85] Augsburger A, Schuchardt A, Hoskins S, Dodd J, Butler S (1999). BMPs as mediators of roof plate repulsion of commissural neurons. Neuron.

[B86] Charron F, Stein E, Jeong J, McMahon AP, Tessier-Lavigne M (2003). The morphogen sonic hedgehog is an axonal chemoattractant that collaborates with netrin-1 in midline axon guidance. Cell.

[B87] Lumsden AG, Davies AM (1983). Earliest sensory nerve fibres are guided to peripheral nerve targets by attractants other than nerve growth factor. Nature.

[B88] Tessier-Lavigne M, Placzek M, Lumsden AG, Dodd J, Jessell TM (1988). Chemotropic guidance of developing axons in the mammalian central nervous system. Nature.

[B89] Guthrie S, Lumsden A (1994). Collagen Gel Coculture of Neural Tissue. Neuroprotocols: A companion to Methods in Neuroscience.

[B90] Cheng HJ, Bagri A, Yaron A, Stein E, Pleasure SJ, Tessier-Lavigne M (2001). Plexin-A3 mediates semaphorin signaling and regulates the development of hippocampal axonal projections. Neuron.

[B91] Kennedy TE, Serafini T, de la Torre JR, Tessier-Lavigne M (1994). Netrins are diffusible chemotropic factors for commissural axons in the embryonic spinal cord. Cell.

[B92] Diaz E, Yang YH, Ferreira T, Loh KC, Okazaki Y, Hayashizaki Y, Tessier-Lavigne M, Speed TP, Ngai J (2003). Analysis of gene expression in the developing mouse retina. PNAS.

[B93] Phillips J, Eberwine JH (1996). Antisense RNA Amplification: A Linear Amplification Method for Analyzing the mRNA Population from Single Living Cells. Methods.

[B94] Bolstad BM, Irizarry RA, Astrand M, Speed TP (2003). A comparison of normalization methods for high density oligonucleotide array data based on variance and bias. Bioinformatics.

[B95] Irizarry RA, Bolstad BM, Collin F, Cope LM, Hobbs B, Speed TP (2003). Summaries of Affymetrix GeneChip probe level data. Nucleic Acids Res.

[B96] Wu Z, Irizarry RA, Gentleman R, Martinez-Murillo F, Spencer F (2004). A Model-Based Background Adjustment for Oligonucleotide Expression Arrays. J Amer Stat Assoc.

[B97] Gautier L, Cope L, Bolstad BM, Irizarry RA (2004). affy--analysis of Affymetrix GeneChip data at the probe level. Bioinformatics.

[B98] Smyth GK, Gentleman R, Carey V, Huber W, Irizarry R, Dudoit S (2005). limma: Linear Models for Microarray Data. Bioinformatics and computational biology solutions using R and Bioconductor.

[B99] Smyth GK (2004). Linear models and empirical bayes methods for assessing differential expression in microarray experiments. Stat Appl Genet Mol Biol.

[B100] Stanford SOURCE. http://source.stanford.edu/.

[B101] NCBI Entrez Gene. http://www.ncbi.nlm.nih.gov/sites/entrez?db=gene.

[B102] Ensembl. http://www.ensembl.org/Mus_musculus/.

[B103] Applied-Biosystems (1997). User Bulletin #2 (04303859).

[B104] GenMAPP - Gene Map Annotator and Pathway Profiler. http://genmapp.org/.

