# Supplementary material for: Analysis of gene expression during neurite outgrowth and regeneration
Source: BMC Neurosci. 2007 Nov 23;8:100. doi: 10.1186/1471-2202-8-100 (PMC2245955; doi:10.1186/1471-2202-8-100)
Supplement: Additional file 1 — Additional Figures and Additional Methods. Contains Additional Figures 1–5, figure legends and Additional Methods. [file 1471-2202-8-100-S1.pdf]

Additional Figure 1

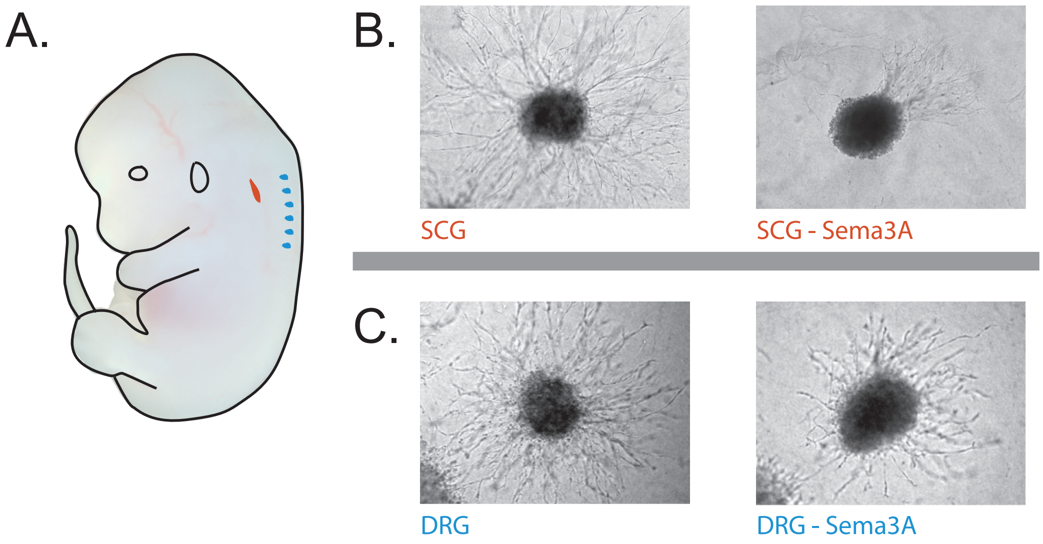

Additional Figure 2

A.

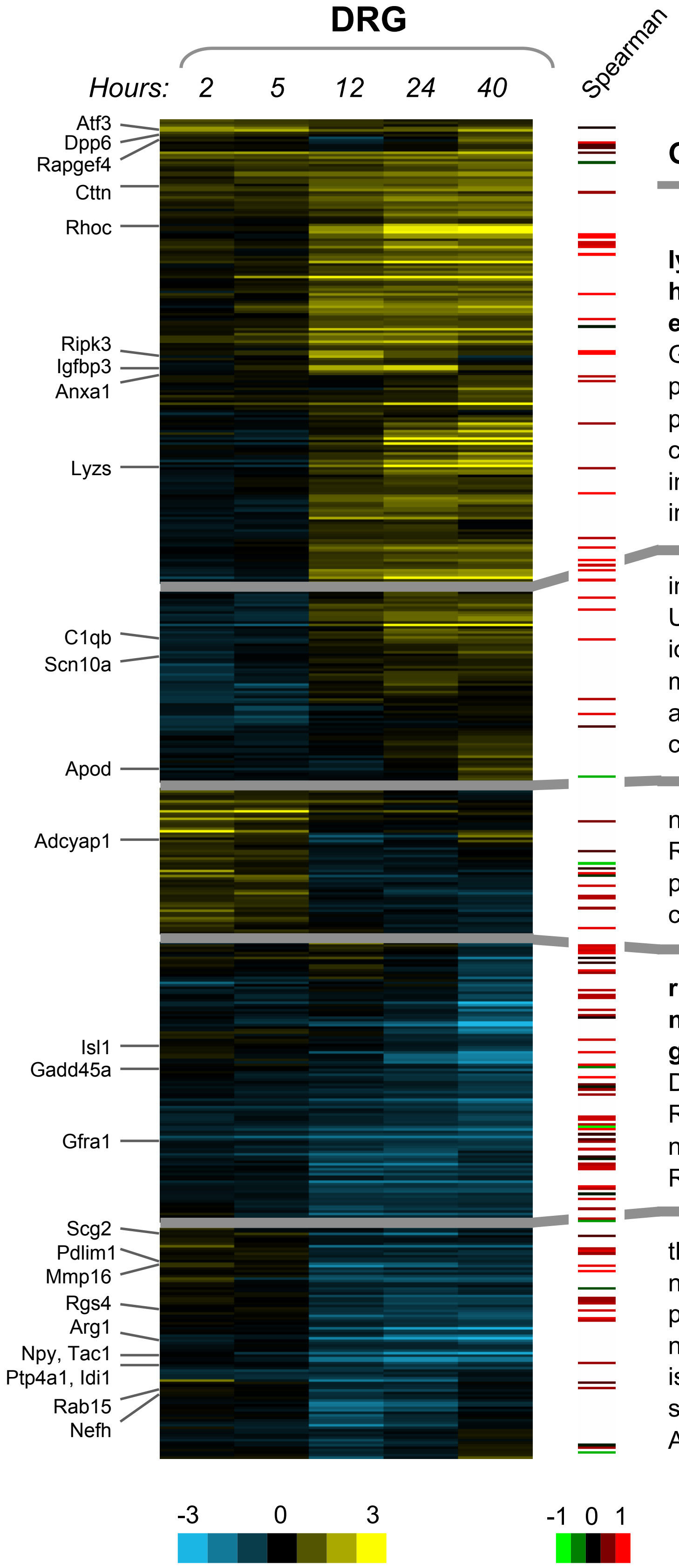

B.

**Gene Ontology category**	changed / measured
**lysosome**	25 / 34
**hydrolase activity - acting on glycosyl bonds**	18 / 23
**extracellular region**	185 / 411
Golgi vesicle transport	11 / 14
peptidase activity	43 / 81
protein amino acid phosphorylation	47 / 97
cellular nerve ensheathment	4 / 4
induction of apoptosis	12 / 20
immune response	29 / 58
integral to membrane	101 / 520
UDP-glycosyltransferase activity	6 / 11
ion transport	27 / 106
membrane lipid biosynthesis	4 / 8
axon extension	3 / 5
cytokine biosynthesis	4 / 8
nucleus	76 / 454
RNA processing	13 / 45
protein ubiquitination	9 / 35
cytoskeleton-dependent intracellular transport	4 / 8
**ribonucleoprotein complex**	28 / 56
**nucleoside metabolism**	7 / 8
**glycolysis**	9 / 12
DNA-directed RNA polymerase II, holoenzyme	5 / 6
RNA export from nucleus	5 / 6
neuropeptide receptor activity	4 / 4
RNA processing	17 / 45
tRNA metabolism	5 / 7
nuclear mRNA splicing - via spliceosome	9 / 19
polyamine biosynthesis	3 / 3
neuron projection	5 / 9
isoprenoid biosynthesis	3 / 4
synaptic transmission	7 / 18
ATP biosynthesis	3 / 5

Additional Figure 3

A.

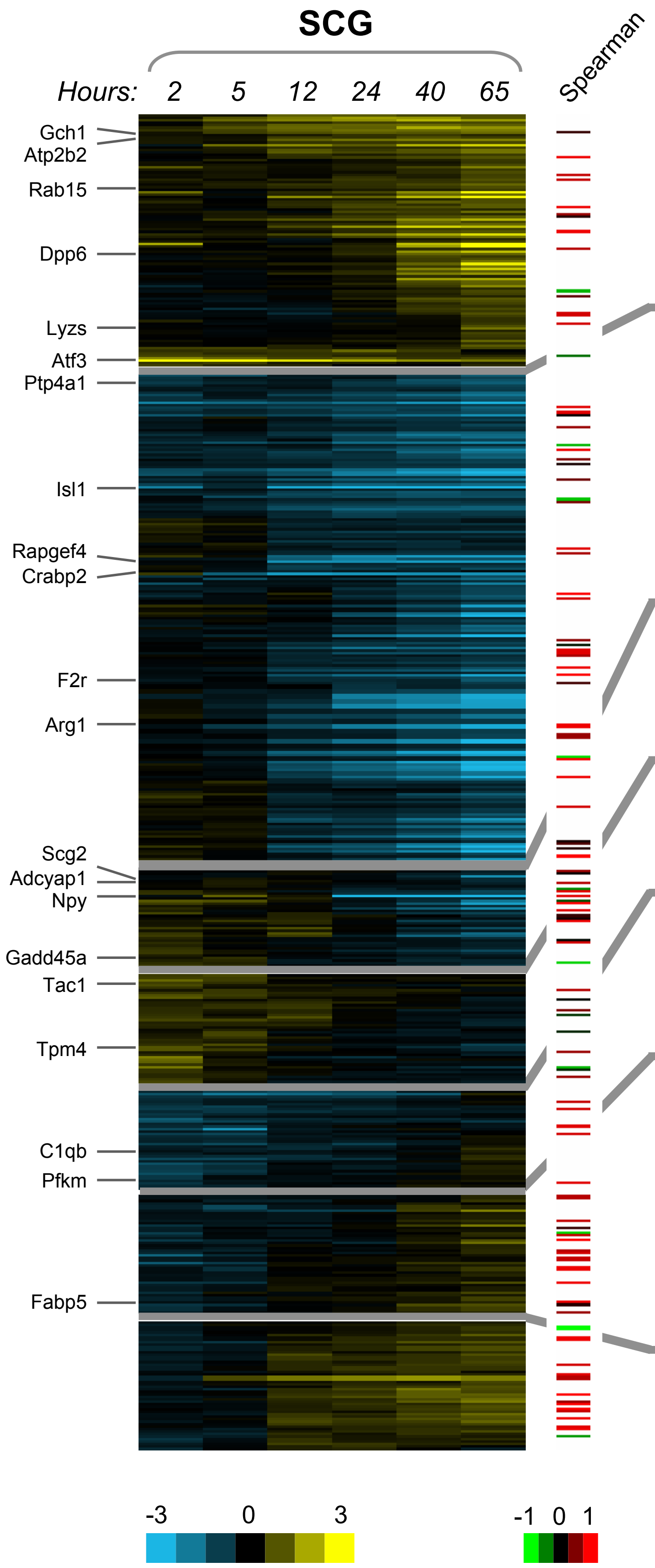

B.

Gene Ontology category	changed / measured
**integral to membrane**	88 / 283
extracellular space	53 / 173
cytoplasm	85 / 314
metal ion transport	13 / 30
establishment of localization	56 / 205
regulation of phosphorylation	4 / 5
vesicle-mediated transport	14 / 38
cholesterol transport	3 / 3
cation binding	59 / 227
ion channel activity	8 / 18
GTPase activity	5 / 9
**nuclear mRNA splicing, via spliceosome**	26 / 35
**nucleic acid binding**	153 / 264
**nucleotide binding**	124 / 243
**mitotic cell cycle**	33 / 40
**DNA replication**	35 / 40
**DNA repair**	26 / 31
**chromosome**	37 / 53
regulation of transcription	78 / 158
protein amino acid phosphorylation	40 / 74
hydrolase activity on acid anhydrides w/ phosphorus	33 / 64
MAP kinase phosphatase activity	3 / 3
chromatin binding	5 / 15
neuropeptide signaling pathway	3 / 8
structural constituent of ribosome	3 / 9
regulation of cell growth	4 / 17
transcription	30 / 165
RNA processing	10 / 55
chromosome organization and biogenesis	8 / 37
signal transducer activity	18 / 125
cell-cell adhesion	3 / 8
oxidoreductase activity	10 / 60
nervous system development	8 / 44
blood vessel morphogenesis	4 / 16
lipid biosynthesis	5 / 25
**biosynthesis**	24 / 92
integral to membrane	46 / 283
carbohydrate metabolism	12 / 44
transporter activity	16 / 75
GTPase activator activity	4 / 9
oxidoreductase activity, paired donors, molecular O_2	5 / 13
tRNA ligase activity	3 / 4
axonogenesis	5 / 15
metal ion binding	40 / 291
cytoskeletal protein binding	7 / 33
**integral to membrane**	64 / 283
actin cytoskeleton organization and biogenesis	6 / 11
**synapse**	7 / 13
lipid metabolism	13 / 55
cofactor metabolism	6 / 16
transferase activity - transferring acyl groups	5 / 14
oxidoreductase activity, acting on CH-OH w/ NAD/P	4 / 11
regulation of neurotransmitter levels	3 / 7

Additional Figure 4

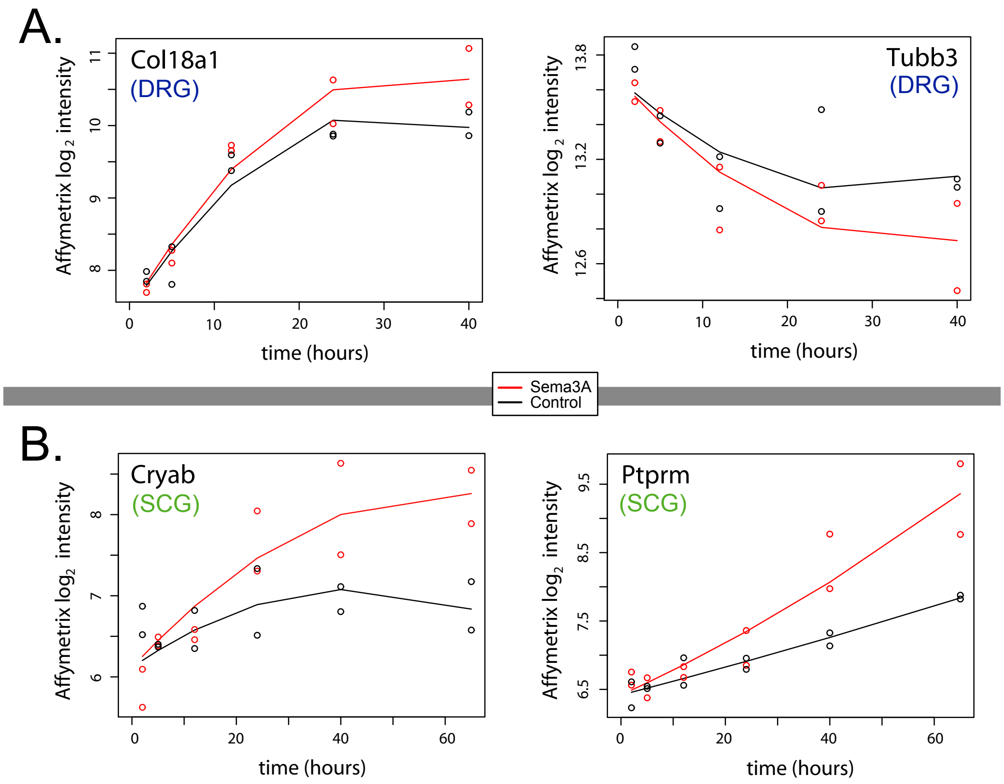

Additional Figure 5

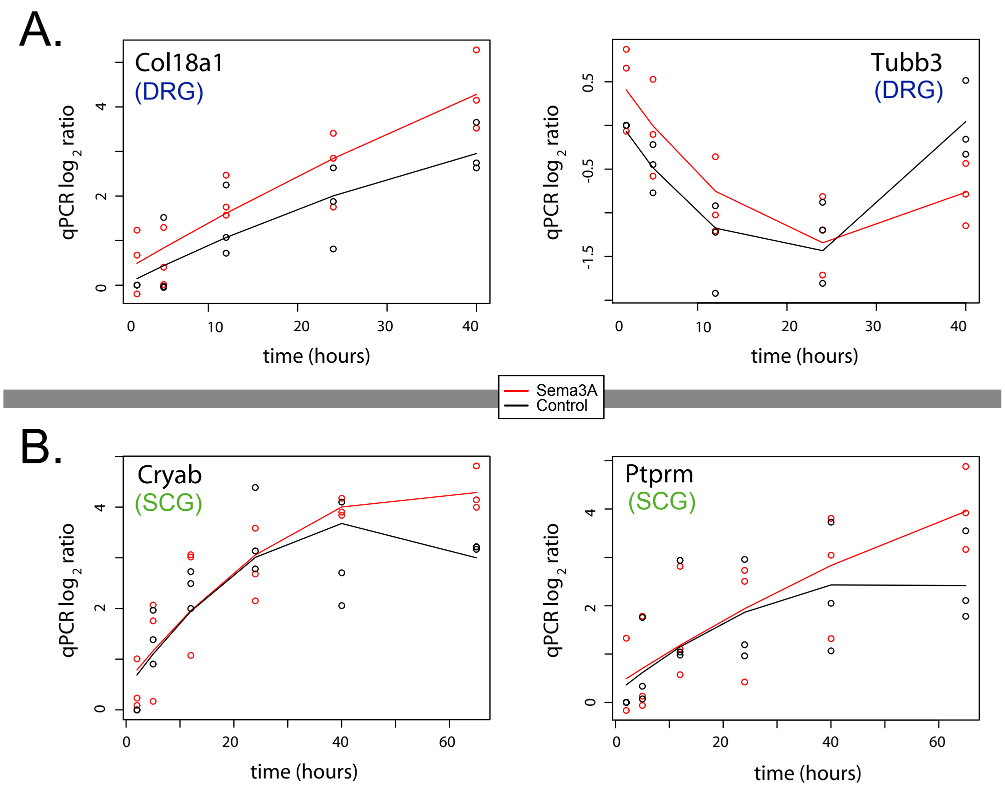

Additional Figures

Additional Figure 1.

Neuronal populations used for the study of neurite outgrowth and guidance during development. Approximate location of SCG (red) and DRG (blue) *in vivo* are shown on a mouse embryo diagram **(A)**. Neuronal explants cultured *in vitro* are also shown at the final time point harvested, with SCG at 65 hours **(B)** and DRG at 40 hours **(C)**. The left panel of each (B,C) shows standard neurite outgrowth, while the right panel shows outgrowth away from a source of Sema3A. Cos7 cells (source of Sema3A) are positioned to the lower left of each explant.

Additional Figure 2.

HOPACH clustering and GenMAPP annotation of genes changed in DRG during neurite outgrowth *in vitro*. **(A)** The cluster displays averaged Affymetrix microarray data for 5 time points of DRG outgrowth (2, 5, 12, 24, and 40 hours), for the 2,632 probe sets found to change more than 1.5 fold ($p \leq 0.05$). Horizontal gray bars separate the first level HOPACH clusters. Genes annotated along the left column are those also found in two or more of the *in vivo* regeneration studies (see Figure 2A; abbreviations listed in Additional Table 1). The far right hand column indicates in a red-green scale the Spearman correlation of 489 genes also tested by spotted (RIKEN) microarrays. Red indicates a positive or high correlation (see color-bar). **(B)** MAPPFinder annotations of Gene Ontology categories represented in the first-level clusters are shown, along with number of genes changed from the total number measured in each category (all Z-score > 2 and permuted-p ≤ 0.05). Terms in bold have a p-value ≤ 0.05 after adjustment for multiple hypothesis testing.

Additional Figure 3.

HOPACH clustering and GenMAPP annotation of genes changed in SCG during neurite outgrowth *in vitro*. **(A)** The cluster displays averaged Affymetrix microarray data for 6 time points of SCG outgrowth (2, 5, 12, 24, 40, and 65 hours), for the 1,728 probe sets found to change more than 1.5 fold ($p \leq 0.05$). Horizontal gray bars separate the first level HOPACH clusters. Genes annotated along the left column are those also found in two or more of the *in vivo* regeneration studies (see Figure 2A; abbreviations listed in Additional Table 1). The far right hand column indicates in a red-green scale the Spearman correlation of 395 genes also tested by spotted (RIKEN) microarrays. Red indicates a positive or high correlation (see color-bar). **(B)** MAPPFinder annotations of Gene Ontology categories represented in the first-level clusters are shown, along with number of genes changed from the total number measured in each category (all Z-score > 2 and permuted-$p \leq 0.05$). Terms in bold have a p-value $\leqq$ 0.05 after adjustment for multiple hypothesis testing.

Additional Figure 4.

Gene expression profiles over time for genes observed by microarray to change during exposure to Sema3A in DRG **(A)** and SCG **(B)**. Individual points and quadratic fit of Affymetrix intensity values (log_2 scale) are plotted on the y-axis, to depict both degree of change over time and also relative level of probeset intensity. These differences between control and Sema-3A treated cultures were statistically significant (quadratic analysis, $p \leq 0.05$), with the exception of Tubb3 ($p = 0.15$), which is shown here for comparison to Figure A5. Gene names are listed in Tables 3-4.

Additional Figure 5.

qPCR did not validate gene expression changes associated with exposure to Sema3A. Plots display qPCR data points and quadratic fit for genes observed in DRG **(A)** and SCG **(B)**. Values are normalized to a common reference gene (Wdr4). Data is expressed in $\log_2$ scale, with the 2 hour control starting point set to zero. Tubb3, with a maximum difference of 1.7-fold between Sema3A and control samples, was the only gene for which a statistically significant difference was observed (quadratic analysis, $p \leq 0.05$). Gene names are listed in Tables 3-4.

Additional Methods

Preparation of spotted microarrays

The RIKEN release I (20K) library was PCR-amplified and spotted on lysine-coated glass slides using a 32-pin DeRisi-style arrayer [1-4]. Spotted microarrays were produced with assistance from the Functional Genomics Lab (FGL) at UC Berkeley and the Core Facility for Genomics and Proteomics at UC San Francisco. Slides were hydrated, pre-washed, and post-processed as per published methods.

cDNA labeling and hybridization for spotted microarrays

Amplified cRNA was labeled using amino-allyl dUTP incorporation, followed by coupling to Cy dye. Labeling and microarray hybridizations were based on published protocols [2, 5]. 1.5 µg cRNA (for SCG samples) or 3 µg (for DRG samples) was mixed with 3 µg of random hexamers. Reverse transcription used SSIII as above, with a 2:3 mix of dUTP to amino-allyl-dUTP. RT reactions were followed by hydrolysis with 10 µl IM NaOH and 10 µl 0.5M EDTA.

Reference pool RNA samples were aliquotted, amplified separately, and then pooled together. To provide a consistent reference pool for a large number of microarray hybridizations, 150 reverse transcription (RT) labeling reactions were carried out per pool. Labeled RTs were also pooled and re-split.

Experimental and reference pool samples were coupled at the same time to either NHS-ester Cy3 or Cy5 dye (Amersham Biosciences) in 3 μl DMSO. Experimental samples were always coupled to Cy5 and reference pool always to Cy3. Overnight hybridization at 50 degrees was done in 25 mM HEPES pH 7.8, 0.5 μg/μl polyA RNA, 3X SSC, 50% formamide, and 0.1% SDS. After hybridization, arrays were washed according to published protocols (see http://microarrays.org). Slides were scanned on a Genepix 4000A scanner using 2 line averaging and 10 micron pixel resolution and saved as single-image TIFFs.

Image analysis and normalization – spotted microarrays

Data were extracted from TIFF images using the Spot 2.0 image analysis package in R 1.7.1. Tolerance was set to ten, with foreground seed size equal to five and crossgap equal to four. Extracted data were written out to tab-delimited Spot files and uploaded into Genetraffic Duo (Iobion) for normalization. Spot files were quality-checked using the SpotTools (maTools) package in Bioconductor [6], and arrays with uneven background or low signal to noise ratio were repeated. Repeat hybridizations were quality-checked again, and the array with better signal to noise ratio was included in the data. Lowess sub-grid normalization was carried out using Genetraffic for all DRG samples as a group and for all SCG samples as a group [7, 8].

Matching genes from Affymetrix and spotted array platforms

Matching of Affymetrix probesets to RIKEN spotted array clones was done using TIGR's Resourcerer (http://www.tigr.org/tigr-scripts/magic/r1.pl) 12.0 July 2005 Release, matching Affy

MOE430A array to the RIKEN 20k set via Entrez Gene (Locuslink). The correlation of array data for matched genes was measured by Spearman correlation in R 2.2.0. Because high background skews the low intensity results of the spotted microarrays, only spots with an median intensity value greater than 256 pixels, or a $\log_2$ intensity value of 8, were included. To deal with multiple RIKEN spots matching to one Affy probeset or vice versa, matches were ranked by intensity and then filtered to include only unique IDs.

References

1. Miki R, Kadota K, Bono H, Mizuno Y, Tomaru Y, Carninci P, Itoh M, Shibata K, Kawai J, Konno H *et al*: **Delineating developmental and metabolic pathways in vivo by expression profiling using the RIKEN set of 18,816 full-length enriched mouse cDNA arrays**. *PNAS* 2001, **98**(5):2199-2204.
2. DeRisi J, Penland L, Brown PO, Bittner ML, Meltzer PS, Ray M, Chen Y, Su YA, Trent JM: **Use of a cDNA microarray to analyse gene expression patterns in human cancer**. *Nat Genet* 1996, **14**(4):457-460.
3. Schena M, Shalon D, Davis RW, Brown PO: **Quantitative monitoring of gene expression patterns with a complementary DNA microarray**. *Science* 1995, **270**(5235):467-470.
4. DeRisi J, Iyer V, Brown PO: **The MGuide: A complete guide to building your own microarrayer**. *http://cmgmstanfordedu/pbrown/mguide* 1998-1999, **Version 2.0**(Biochemistry Department, Stanford University, University of California San Francisco).
5. Diaz E, Yang YH, Ferreira T, Loh KC, Okazaki Y, Hayashizaki Y, Tessier-Lavigne M, Speed TP, Ngai J: **Analysis of gene expression in the developing mouse retina**. *PNAS* 2003, **100**(9):5491-5496.
6. Dudoit S, Gentleman RC, Quackenbush J: **Open source software for the analysis of microarray data**. *Biotechniques* 2003, **Suppl**:45-51.
7. Smyth GK, Speed T: **Normalization of cDNA microarray data**. *Methods* 2003, **31**(4):265-273.
8. Yang YH, Dudoit S, Luu P, Lin DM, Peng V, Ngai J, Speed TP: **Normalization for cDNA microarray data: a robust composite method addressing single and multiple slide systematic variation**. *Nucl Acids Res* 2002, **30**(4):e15-.
